# Characterizing amblyopic perception under non-rivalrous viewing conditions

**DOI:** 10.1038/s41598-023-31301-8

**Published:** 2023-05-17

**Authors:** Kimberly Meier, Kristina Tarczy-Hornoch, Geoffrey M. Boynton, Ione Fine

**Affiliations:** 1 Department of Psychology, University of Washington, Seattle, WA USA; 2 Department of Ophthalmology, University of Washington, Seattle, WA USA

**Keywords:** Visual system, Pattern vision

## Abstract

Current assessments of interocular interactions in amblyopia often use rivalrous stimuli, with conflicting stimuli in each eye, which does not reflect vision under typical circumstances. Here we measure interocular interactions in observers with amblyopia, strabismus with equal vision, and controls using a non-rivalrous stimulus. Observers used a joystick to continuously report the perceived binocular contrast of dichoptic grating stimuli, identical except that the stimulus was contrast-modulated independently in each eye over time. Consistent with previous studies, a model predicting the time-course of perceived contrast found increased amblyopic eye attenuation, and reduced contrast normalization of the fellow eye by the amblyopic eye, in amblyopic participants compared to controls. However, these suppressive interocular effects were weaker than those found in previous studies, suggesting that rivalrous stimuli may overestimate the effects of amblyopia on interocular interactions during naturalistic viewing conditions.

## Introduction

Amblyopia is a visual disorder, clinically characterized as poor visual acuity in one eye even with best optical correction in place. Amblyopia, which affects 1–2% of the population^1^, arises when a child experiences abnormal vision in one eye for a prolonged period of development, typically because of anisometropia (unequal refractive error in the two eyes), strabismus (an eye misalignment), or a combination of the two, resulting in subnormal best-corrected vision^2^. Amblyopia is a consequence of the abnormal progression of cortical development in response to this atypical visual input during key periods of maturation^3^, rather than being directly attributable to optical or physical characteristics of the eye.

Amblyopia results in disruption of a variety of visual functions routinely assessed in the clinic, such as acuity, contrast sensitivity, fusion, and stereopsis (reviewed in ^4–6^). Generally, the mechanisms underlying binocular amblyopic deficits are thought to include attenuation of the amblyopic eye signal and abnormal interocular suppression, or inhibition, of the signal of one eye by the other, often modelled as some form of gain control^7,8^, e.g. contrast normalization^9–11^.

A wide variety of dichoptic stimuli^12^ have been used to characterize the effects of amblyopia on binocular vision, including gratings and plaids^8,13–15^, moving dots^16^, and letters^17^. However, these tasks have all relied on dichoptic images that differ in their spatial content, orientation, signal/noise properties, phase etc. across the two eyes. These stimuli are generally rivalrous or quite difficult to fuse (as is the case for phase disparities, especially for controls).

**Figure 1:**
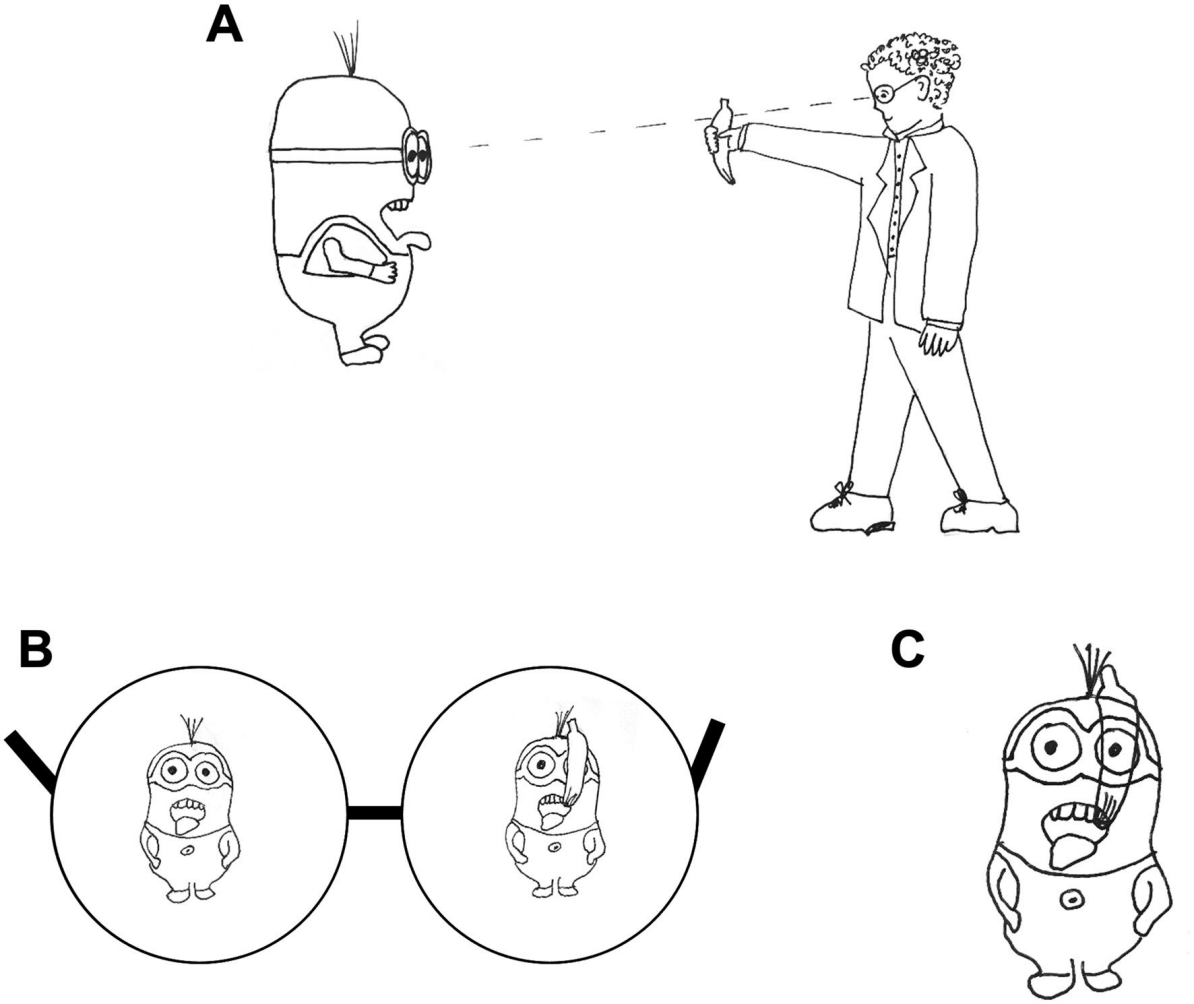
In typical binocular vision (**A**) most of the visual scene is consistent between the left and right eye (**B**, the region of Dave the Minion’s overalls), and the statistically optimal strategy is to combine signals from the two eyes with a weighting that reflects the relative ‘efficiency’ of each eye. However in certain circumstances (e.g. partial visual occlusion by a banana) the input received by the two eyes will differ substantially over a local region of the scene (Dave’s goggles) and any weighted average of the two eyes will be perceptually nonsensical (**C**). In these regions of the scene the best strategy is a ‘winner-take-all’ where one eye’s input dominates.

One concern with this approach is that rivalrous conditions do not reflect the naturalistic viewing conditions experienced in daily life. When both eyes are open (assuming adequate correction of etiological factors, such as proper spectacle prescription and surgical alignment), the visual cortex receives consistent input from each eye over most of the visual scene, as shown in Fig. 1A. In regions where the visual scene is consistent across the two eyes, the statistically optimal strategy is presumably to combine signals from the two eyes with a weighting that reflects the relative ‘reliability’ of each eye. However, there are circumstances (e.g. differential occlusion in each eye of a distant object by a nearer object), Fig. 1B, where the input between the two eyes differs substantially over a local region of the scene, and any weighted average of the two eyes will be perceptually nonsensical, Fig. 1C. In these rivalrous regions, the best strategy is presumably a ‘winner-take-all’ in which the input from the most ‘reliable’ eye dominates. Thus, the mechanisms of attenuation and interocular suppression that operate under conditions of rivalry—as used for most measures of interocular suppression in amblyopia—might easily be very different from those that underlie neural responses under more typical non-rivalrous viewing conditions.

To address this, we developed a non-rivalrous task for assessing the neural mechanisms underlying amblyopia. Participants were shown dichoptically-presented grating stimuli whose spatial content was identical, except for their contrast, which was modulated independently in each eye over time. Participants dynamically adjusted the position of a joystick lever over time to match their perception of stimulus contrast.

To maximize our ability to examine interocular interactions, we used 2 cpd grating stimuli. This is roughly the peak of the contrast sensitivity function for amblyopic eyes^18^; indeed, amblyopic observers often do not show interocular differences in monocular contrast detection thresholds at this spatial frequency^19^. Thus our choice of a relatively low spatial frequency stimulus was designed to ensure that our measurements primarily reflected suprathreshold interocular interactions^20^ rather than monocular attenuation from a degraded input signal.

We found that, for our non-rivalrous stimulus, a simple model based on *monocular* *attenuation* *in* *the* *context* *of* *binocular* *stimulation* (k_AE_), and *interocular* *contrast* *normalization* (μ_AE_: normalization of the amblyopic by the fellow eye; μ_FE_: normalization of the fellow by the amblyopic eye) could successfully predict response time-courses. Fitted model parameters predicted performance on independent clinical measures of stereoacuity and the interocular balance point.

Our estimates of interocular contrast normalization, as measured in our naturalistic task, were considerably weaker than those previously reported for rivalrous conditions, suggesting that the ‘perceptual burden’ of amblyopia, as measured under naturalistic viewing conditions, may be weaker than has previously been supposed.

## Results

### Dynamic contrast estimation task

**Figure 2:**
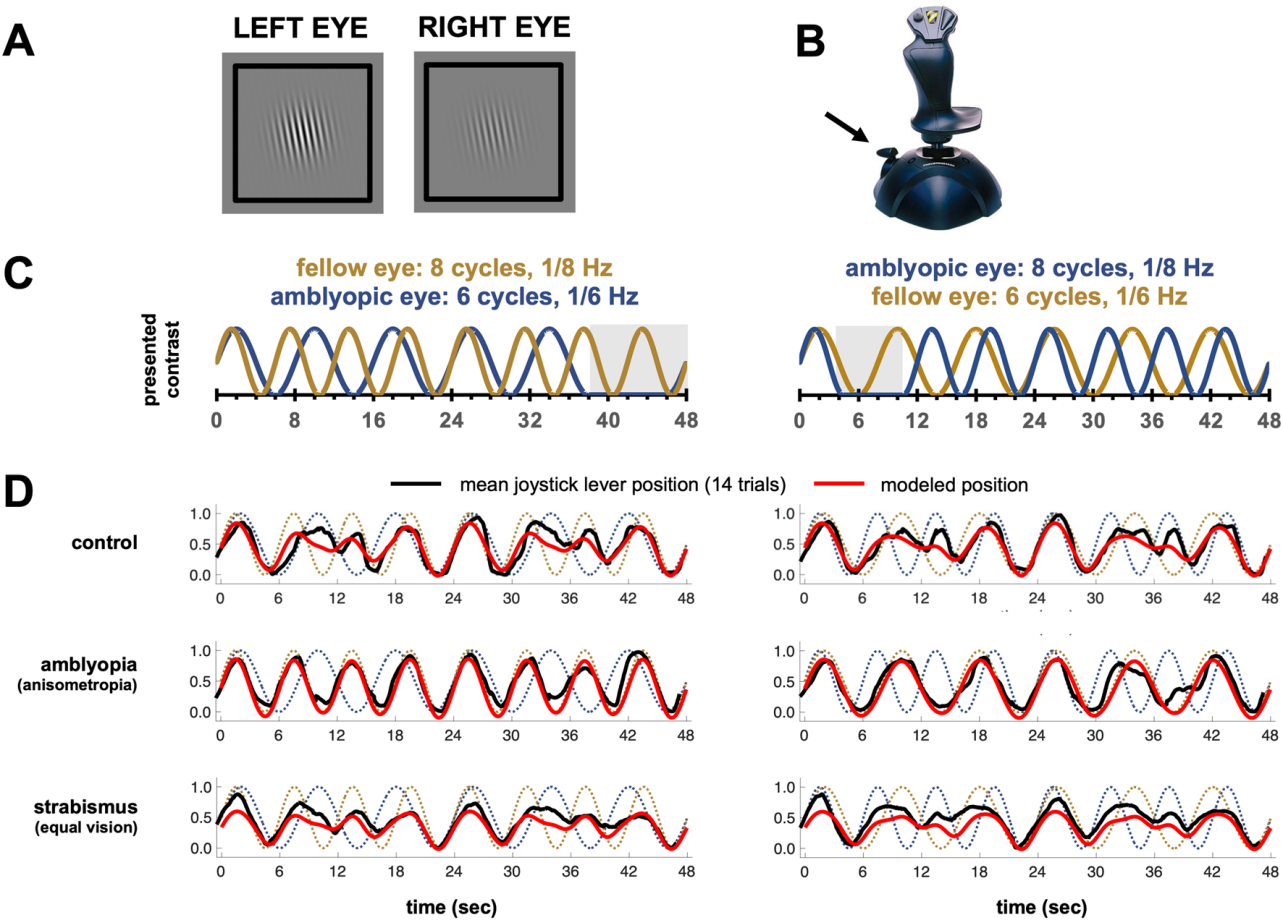
(**A**) Example dichoptic stimulus. (**B**) Participants reported binocular perceived contrast using their thumb and forefinger to move the throttle lever of a joystick. (**C**) Trial schematic indicating contrast modulation in each eye over time. In half of the trials the fellow eye contrast modulated at 1/8 Hz and amblyopic eye at 1/6 Hz (left panel) or vice versa (right panel). Example monoptic portions embedded in the trial are shown in grey, these regions differed across each trial. (**D**) Example mean time-courses averaged across all 14 trials per condition (dichoptic periods only), with model fits (based on the full dataset), for typical participants from the control, amblyopic and non-amblyopic strabismus with equal vision groups.

The stimulus, shown in Fig. 2A, consisted of a Gaussian-windowed 2 cpd grating on a mean-luminance grey background. The grating rotated slowly counter-clockwise at 1°/s for the duration of the trial to minimize adaptation. Each trial began with 14 s of *binocular* 1/7 Hz contrast modulation, in which the contrast of the two eyes was identical. Most of the remaining 48 s of the trial was *dichoptic*, such that contrast in one eye modulated at 1/6 Hz while the contrast in the other eye modulated at 1/8 Hz (or vice versa). The periods of these sinusoidal modulations synchronized every 24 s, so each modulation pattern was repeated once during each trial. Embedded in each trial was a short phase of *monoptic* stimulation in which the contrast modulation in one eye ‘dropped out’ and remained at 0% contrast for a single cycle (6 or 8 s), while the other eye continued to modulate. Participants reported perceived contrast using the throttle lever (Fig. 2B) of a Thrustmaster USB Joystick (Guillemot Corporation SA). Example contrast time-courses are shown for two trials in Fig. 2C.

We used a three-stage model fitting procedure to predict joystick position over time as a function of the contrast presented to each eye. The first stage calibrated the relationship between joystick position and perceived binocular contrast using the portion of each trial that was binocular, the second stage modeled monocular attenuation using the portion of each trial that was monoptic, and the third stage modeled binocular interactions using the portions of each trial that were dichoptic.

Our model was adapted from a binocular normalization model^11^ originally built to describe interocular contrast suppression as measured by BOLD responses in V1. This model was selected primarily for its simplicity and its ability to clearly delineate attenuation and suppression with separable parameters. As described in the Supplementary Materials, for our data this model performs similarly to previous similar and more complex models in the literature^7–10^. However it is important to note that our stimuli were designed to efficiently measure attenuation and normalization under non-rivalrous conditions, not to differentiate between different models. Thus, it is not surprising that, for the stimuli used in our experiment, all the models we examined make relatively similar predictions, see Supplementary Materials, Appendix I.

Figure 2D displays the mean joystick lever position over time averaged over all 14 trials for typical individuals from each group. The dotted lines show the contrasts presented to each eye, the red lines show the model’s predicted joystick position, and the black lines show actual joystick position. Both conditions (contrast modulation 1/8 Hz amblyopic, 1/6 Hz fellow; and 1/6 in amblyopic, 1/8 Hz fellow) are shown. The model accurately predicts perceived contrast over time. Inspection of residuals revealed no obvious systematic deviations between model predictions and joystick position. Example-course data for single trials is shown for the same individual participants in Supplementary Fig. 1.

### Modeling stage 1: joystick vs. contrast calibration

The first modeling stage characterized idiosyncratic deviations between joystick position and presented binocular contrast using a simple transformation between stimulus contrast and joystick position, fit to data from the binocular trial phase only. This transformation consisted of a parameterized static linear function and a short delay between the stimulus contrast and joystick position. Inverting this transformation maps joystick position to perceived binocular contrast, which we call the calibrated joystick position. Best-fitting delay and linear function parameters were found by minimizing the mean square error (MSE) between the calibrated joystick position and stimulus contrast *C* as a function of time. None of the joystick calibration parameter values, including the mean squared error of the calibration fit, differed significantly between groups, see Supplementary Table 1.

### Stage 2: monocular attenuation

Next, data from the monoptic phase of each trial were used to estimate the monocular attenuation weights k_L_ and k_R_, as follows:

1 $$\widehat{C}(t)= \left({k}_{R}{C}_{R}\left(\mathrm{t}\right)+ {k}_{L}{C}_{L}(\mathrm{t})\right)/ \mathrm{max}({k}_{R}, {k}_{L})$$

where $\widehat{C}\left(t\right)$ is the model prediction of equivalent perceived binocular contrast, and ${C}_{R}\left(\mathrm{t}\right)$ and ${C}_{L}(\mathrm{t})$ are the contrasts of the stimuli presented to right and left eyes respectively. Because this model was only fit to the monoptic phase of the trials, either ${C}_{R}\left(\mathrm{t}\right)$ or ${C}_{L}(\mathrm{t})$ was always 0. The normalization by $\mathrm{max}({k}_{R}, {k}_{L})$ meant that either ${k}_{R}$ or ${k}_{L}$ became 1. We defined the eye with k = 1 as the ‘fellow’ eye, and the eye with k < 1 as the ‘amblyopic’ eye, ${k}_{AE}$, in all observers, with ${k}_{AE}$ characterizing linear monocular attenuation in the amblyopic eye. This model is a reduced form of the full model, as described below, with μ_R_ = μ_L_ = 0, and σ = 1.

**Figure 3:**
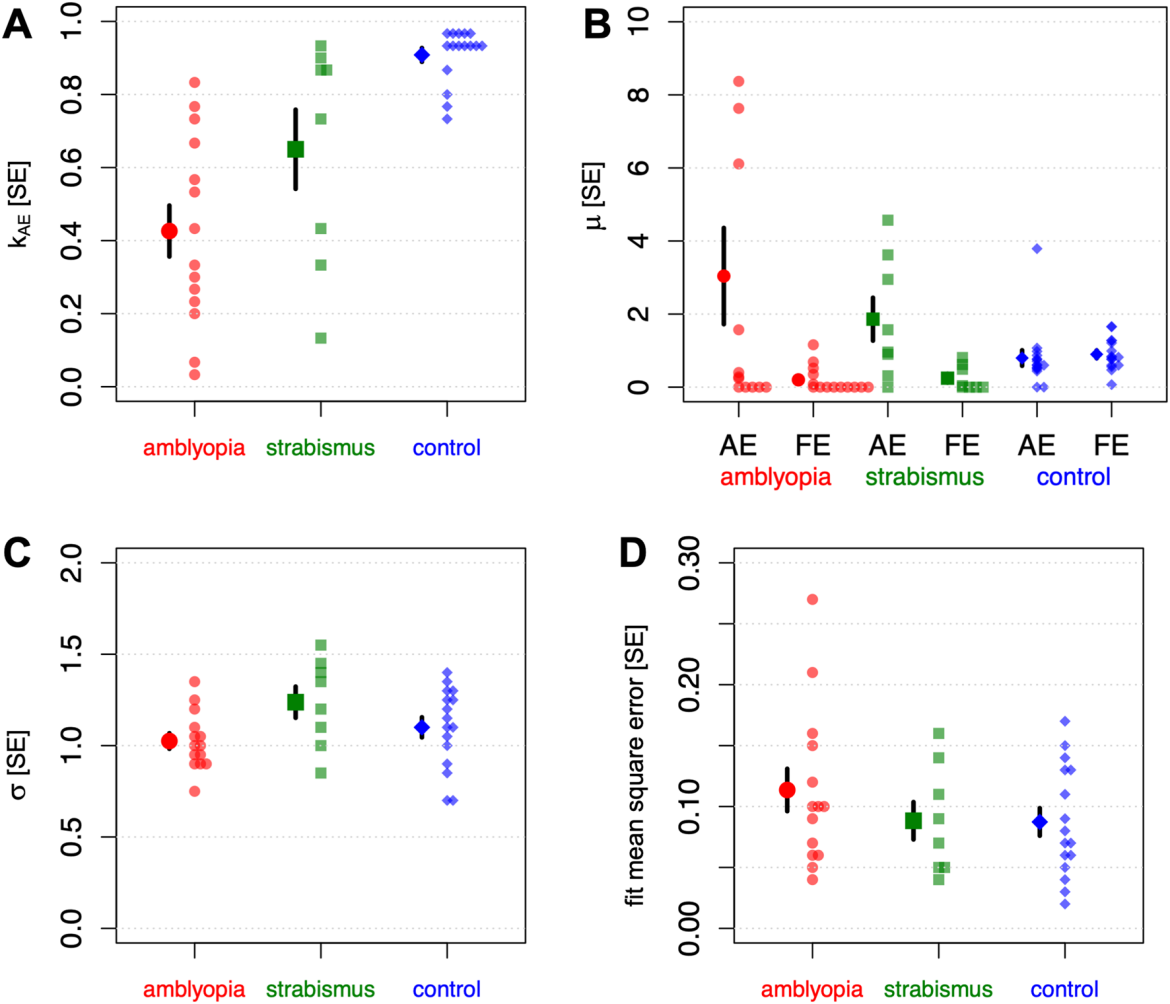
Parameter values used to fit the dynamic contrast estimation task. (**A**) k_AE_, (**B**) μ_AE_ and μ_FE_, (**C**) σ, (**D**) MSE. Mean values and single standard errors are shown with the larger symbols, individual data points are shown with the smaller symbols. Subjects in the amblyopia group had strabismic, anisometropic or combined amblyopia. Subjects in the strabismus group had equal visual acuity.

As shown in Fig. 3A and Table 1, ${k}_{AE}$ was significantly lower in observers with amblyopia than in controls (*p* < 0.0001), though it is worth noting that there was significant individual variability in participants with amblyopia. In strabismus with equal acuity, values of ${k}_{AE}$ were intermediate between individuals with amblyopia and controls.

**Table 1:** Monocular attenuation and binocular interactions.

	Control M (SD)	Amblyopia M (SD)	Strabismus with equal acuity M (SD)
k_AE_	0.91 (0.08)	0.42 (0.26) *	0.65 (0.30)
	*t*(14.9) = 6.68, *p* < 0.0001; *Glass’s* *∆* = 6.39		
μ_AE_	0.80 (0.85)	3.04 (4.75)	1.86 (1.66)
μ_FE_	0.90 (0.43)	0.20 (0.36) *	0.24 (0.34)
	No main effects of group *F*(1,27) = 1.77, *p* = 0.20, ${\eta }_{G}^{2}$ = 0.03; main effect of eye, *F*(1,27) = 4.70, *p* = 0.039, ${\eta }_{G}^{2}$ = 0.085; a significant group by eye interaction, *F*(1,27) = 5.42, *p* = 0.028, ${\eta }_{G}^{2}$ = 0.097. Simple main effects: no difference in amblyopic eye, *F*(1,27) = 3.46, *p* = 0.074, ${\eta }_{G}^{2}$ = 0.11; difference in fellow eye *F*(1,28) = 22.98, *p* < 0.0001, ${\eta }_{G}^{2}$ = 0.45.		
σ	1.10 (0.22)	1.02 (0.16)	1.23 (0.23)
	*t*(27.3) = 1.11, *p* = 0.28; *Glass’s* *∆* = 0.36		
MSE	0.087 (0.045)	0.114 (0.065)	0.090 (0.043)
	*t*(22.8) = 1.26, *p* = 0.20; *Glass’s* *∆* = 0.58		

Mean (SD) values are shown.

*Statistically significant difference between amblyopia and control groups.

### Stage 3: binocular interactions

We then modeled the remaining dichoptic portions of each trial, using a simple model, consisting of attenuation (described by the parameter ${k}_{AE}$ estimated in Stage 2), divisive normalization and the linear combination of signals from the two eyes. Initially, the parameter ${k}_{AE}$ was held fixed, having been estimated from the monocular intervals as described above. After estimation of σ, ${\upmu }_{AE}$, and ${\upmu }_{FE}$, these parameters were held constant and the value of ${\mathrm{k}}_{AE}$ was re-estimated using the full dataset (i.e. both monoptic and dichoptic phases).

2 $${\widehat{C}}_{AE}\left(\mathrm{t}\right)= \frac{{k}_{AE}{C}_{AE}\left(t\right)}{{\mu }_{AE}{C}_{FE}\left(\mathrm{t}\right)+\sigma }; {\widehat{C}}_{FE}(\mathrm{t})= \frac{{C}_{FE}(\mathrm{t}) }{{\mu }_{FE}{.k}_{AE}{C}_{AE}\left(t\right)+\sigma }$$

Thus ${\upmu }_{AE}$ and ${\upmu }_{FE}$ reflect the extent to which the signal in the designated eye is reduced, or suppressed, by the signal in the other eye; in a ‘perfectly balanced’ system we would expect μ_AE_ = μ_FE_.

Finally, we assume the final perceived contrast is simply the sum of the normalized attenuated values for the left and right eyes (no additional free parameters).

3 $$\widehat{C}(t)= {\widehat{C}}_{AE}(\mathrm{t})+ {\widehat{C}}_{FE}(t)$$

As shown in Fig. 3B, and Table 1, there was a significant group by eye interaction (*p* = 0.028), driven by significantly lower μ_FE_ (less suppression of the fellow by the amblyopic eye) in observers with amblyopia compared to controls (*p* < 0.0001): suggesting that the amblyopic eye contributed weakly to contrast normalization in the fellow eye. Interestingly, nine participants with amblyopia (64%) and four (50%) participants with strabismus have values of zero or near-zero for μ_FE_, a phenomenon not demonstrated to the same extent by participants in the other groups. μ_AE_ (suppression of the amblyopic by the fellow eye) was not statistically different between the groups (*p* = 0.074). Thus, in our model the effects of amblyopia on binocular interactions were predominantly described by the amblyopic eye failing to contribute to contrast normalization in the fellow eye, rather than an increase in normalization from the fellow eye to amblyopic eye, though many participants with amblyopia also obtained near-zero values for this parameter (five, or 36%), unlike the strabismus-only group.

One consideration was that (despite being fit using a process designed to minimize trade-offs) attenuation and suppression might play a similar role, and trade-off against each other in the model. However the correlation between attenuation (${\mathrm{k}}_{AE}$) and suppression (μ_AE_) across all participants was *r* = −0.17, *t*(36) = 1.03, *p* = 0.31; and within the amblyopia group only, this was *r* = 0.23, *t*(12) = 0.78, *p* = 0.45. Thus, it does not appear that *less* suppression of the amblyopic eye occurs when it is already highly attenuated, as would occur if these parameters traded off against each other in the model.

Finally, neither the saturation constant (σ) or the model MSE differed across control and amblyopia groups, Fig. 3C,D, and Table 1; observers with non-amblyopic strabismus had similar values.

### Assessments of visual function

**Figure 4:**
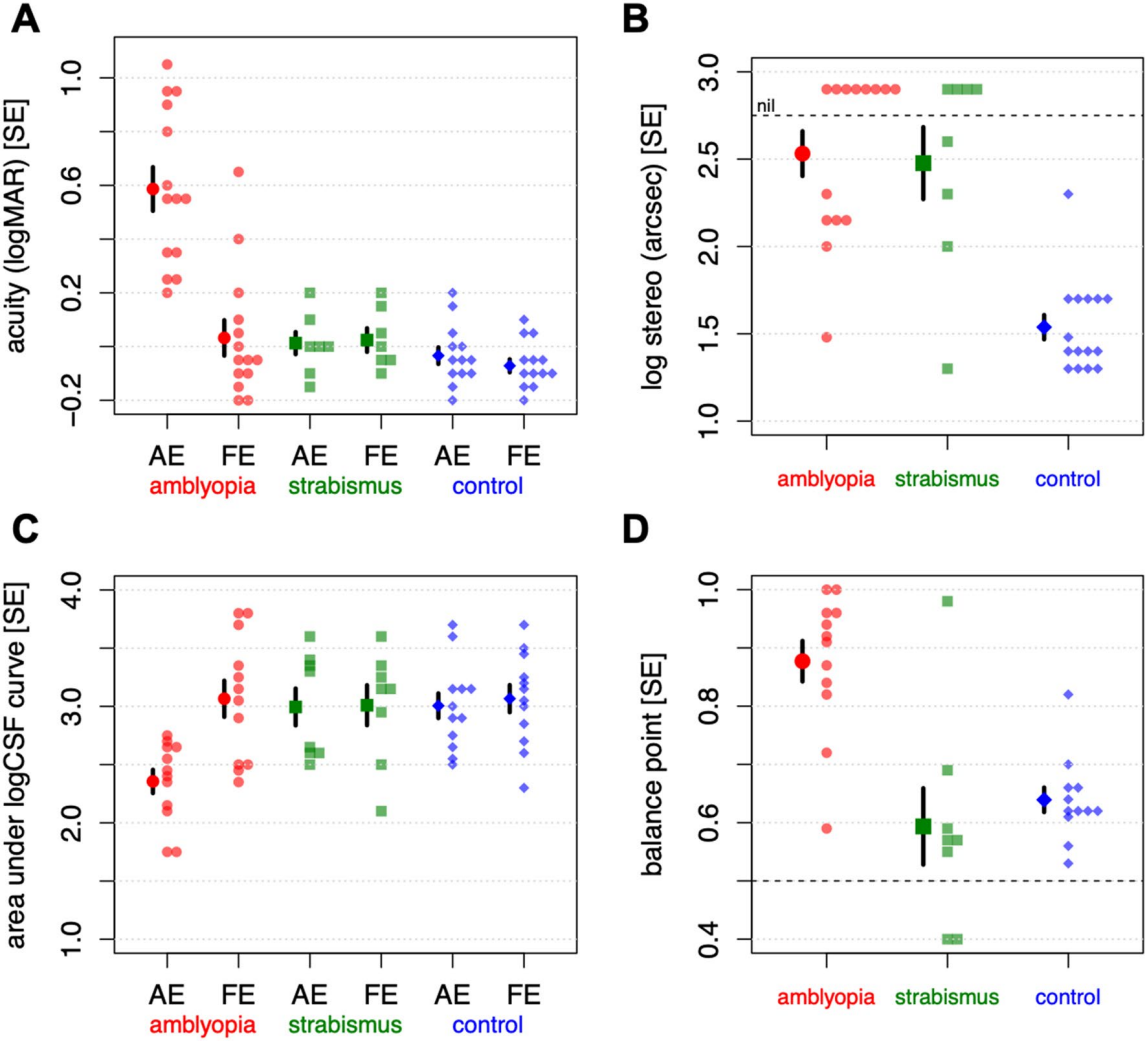
Measures of visual performance. (**A**) Acuity, (**B**) stereo, (**C**) area under log CSF curve and (**D**) interocular balance point. Mean values and single standard errors are shown with the larger symbols, individual data points are shown with the smaller symbols. Subjects in the amblyopia group had strabismic, anisometropic or combined amblyopia. Subjects in the strabismus group had equal visual acuity.

In addition to the dynamic contrast task described above, we carried out four assessments of visual function, as shown in Fig. 4 and Table 2.

**Table 2:** Results for assessments of visual function.

		Control M (SD)	Amblyopia M (SD)	Strabismus with equal acuity M (SD)
Monocular visual acuity (logMAR)	Amblyopic	−0.03 (0.11)	0.59 (0.30) *	0.01 (0.11)
	Fellow	−0.07 (0.09)	0.03 (0.25)	0.02 (0.12)
	Statistics	Main effect of group, *F*(1,25) = 33.72, *p* < 0.0001, ${\eta }_{G}^{2}$ = 0.44; Main effect of eye, *F*(1,25) = 31.81, *p* < 0.0001, ${\eta }_{G}^{2}$ = 0.35; eye by group interaction, *F*(1,25) = 24.22, *p* < 0.0001, ${\eta }_{G}^{2}$ = 0.29. Simple main effects: difference in the amblyopic eye, *F*(1,25) = 47.85, *p* < 0.0001, ${\eta }_{G}^{2}$ = 0.66, no difference in the fellow eye, *F*(1,25) = 2.05, *p* = 0.16, ${\eta }_{G}^{2}$ = 0.08		
Binocular visual acuity (logMAR)	Binocular	−0.13 (0.07)	0.01 (0.17) *	−0.04 (0.12)
		*t*(18.08) = 2.70, *p* = 0.015; *Glass’s* *∆* = 1.88		
Binocular summation (acuity_bino_ – acuity_best mono_)	Compares monocular vs. binocular acuity	0.05 (0.05) *t*(11) = 2.94, *p* = 0.013	0.02 (0.12) *t*(13) = 0.76, *p* = 0.46	0.03 (0.04) t(6) = 2.40, p = 0.053
		*t*(19.18) = 0.66, *p* = 0.51; *Glass’s* *∆* = 0.42		
Randot preschool circles stereopsis (log10 arcsec)		1.54 (0.27) 35 arcsec	2.56 (0.47) * 360 arcsec	2.48 (0.58) 300 arcsec
		*t*(22.25) = 7.25, *p* < 0.0001; *Glass’s* *∆* = 3.68		
Contrast sensitivity: area under the log CSF	Amblyopic	3.01 (0.37)	2.36 (0.35) *	3.00 (0.45)
	Fellow	3.07 (0.40)	3.07 (0.54)	3.01 (0.49)
		Main effect of group, *F*(1,22) = 4.53, *p* = 0.045, ${\eta }_{G}^{2}$ = 0.14; Main effect of eye, *F*(1,22) = 23.78, *p* < 0.0001, ${\eta }_{G}^{2}$ = 0.19; Eye by group interaction, *F*(1,22) = 16.86, *p* < 0.0001, ${\eta }_{G}^{2}$ = 0.14. Simple main effects: difference in the amblyopic eye, *F*(1,23) = 20.85, *p* < 0.0001, ${\eta }_{G}^{2}$ = 0.47, no difference in the fellow eye, *F*(1,23) = 0.00, *p* = 0.98, ${\eta }_{G}^{2}$ = 0.00		
Contrast sensitivity at 2 cpd	Amblyopic	1.97 (0.17)	1.91 (0.34)	1.92 (0.30)
	Fellow	1.91 (0.24)	1.88 (0.25)	1.91 (0.30)
		No effect of condition, *F*(1,22) = 0.24, *p* = 0.629, ${\eta }_{G}^{2}$ = 0.01; no effect of eye, *F*(1,22) = 0.67, *p* = 0.42, ${\eta }_{G}^{2}$ = 0.01; No eye by condition interaction, *F*(1,22) = 0.05, *p* = 0.83, ${\eta }_{G}^{2}$ = 0.00		
Interocular balance point	Binocular interactions	0.64 (0.07)	0.88 (0.12) *	0.59 (0.19)
		*t*(18.10) = 5.85, *p* < 0.0001; *Glass’s* *∆* = 3.26		

*Statistically significant difference between amblyopia and control groups.

#### Monocular and binocular acuity

Amblyopia participants had significantly worse monocular acuity than controls in the amblyopic (*p* < 0.0001), but not the fellow eye (*p* = 0.16), Fig. 4A. Although binocular visual acuity was within normal range for observers with amblyopia, measured values were significantly lower than those of control participants (*p* = 0.015). Acuity in observers with non-amblyopic strabismus resembled controls.

Binocular summation (or inhibition) of acuity is defined as the increase (or decrease) in acuity when using both eyes, relative to the best monocular visual acuity. Control observers showed binocular summation significantly different from zero (*p* = 0.031), while participants with amblyopia and those with strabismus with equal vision showed no significant change in acuity (in either direction) with binocular viewing (*p* = 0.50 and 0.053, respectively). However, the degree of summation in the control group did not differ significantly from that in the amblyopia group (*p* = 0.51).

#### Stereopsis

Stereoacuity on the Randot Preschool Circles test is shown in Fig. 4B; stereoacuity was significantly worse in observers with amblyopia than controls (*p* < 0.0001). Stereoacuity in strabismus with equal vision resembled the amblyopia group.

#### Contrast sensitivity

There were no differences in sensitivity at 2 cpd (group: *p* = 0.24; eye: *p* = 0.42; interaction: *p* = 0.83), confirming no group differences in contrast sensitivity at the spatial frequency of our stimulus.

Area under the log contrast sensitivity curve (AUC) is shown in Fig. 4C. Reflecting acuity losses, participants with amblyopia had a significantly smaller AUC in the amblyopic (*p* < 0.0001) but not the fellow eye (*p* = 0.98) compared to controls, due to lower sensitivity to higher spatial frequencies in the amblyopic eye. Individuals with strabismus showed sensitivity on par with controls.

#### Interocular balance point

The interocular balance point was assessed by finding the relative contrast a participant requires to report seeing the letters in the left and right eye with equal probability^17,21^. A typical control observer will have a balance point of about 0.5, indicating an equal amount of contrast is necessary in each eye, and a typical observer with amblyopia will have a balance point greater than 0.5, for example, a balance point of 0.7 indicates 70% contrast in the amblyopic eye is necessary for an observer to be equally likely to report the letters shown to the amblyopic and fellow eye.

Amblyopic observers had balance points that were significantly higher than those of control observers (*p* < 0.0001), Fig. 4D. Within participants with amblyopia, there was no significant relationship between balance point and acuity, *r* = 0.12, *t*(10) = 0.38, *p* = 0.72, indicating these balance point values are not simply a function of reduced acuity in the amblyopic eye. All but one participant with strabismus and equal acuity had balance point values within the control range.

#### Summary

In summary, our clinical determination of control, amblyopia and strabismus with equal acuity were reflected in functional tests of visual performance. Participants with amblyopia showed reduced acuity in the amblyopic eye (unsurprisingly, given that they were clinically identified based on acuity), lower contrast sensitivity, shifted interocular balance and reduced stereopsis. Participants classified as non-amblyopic strabismus with equal acuity showed unimpaired acuity (by definition), contrast sensitivity and interocular balance, but did show reduced stereopsis.

### The relationship between model parameters and assessment of visual function

**Figure 5:**
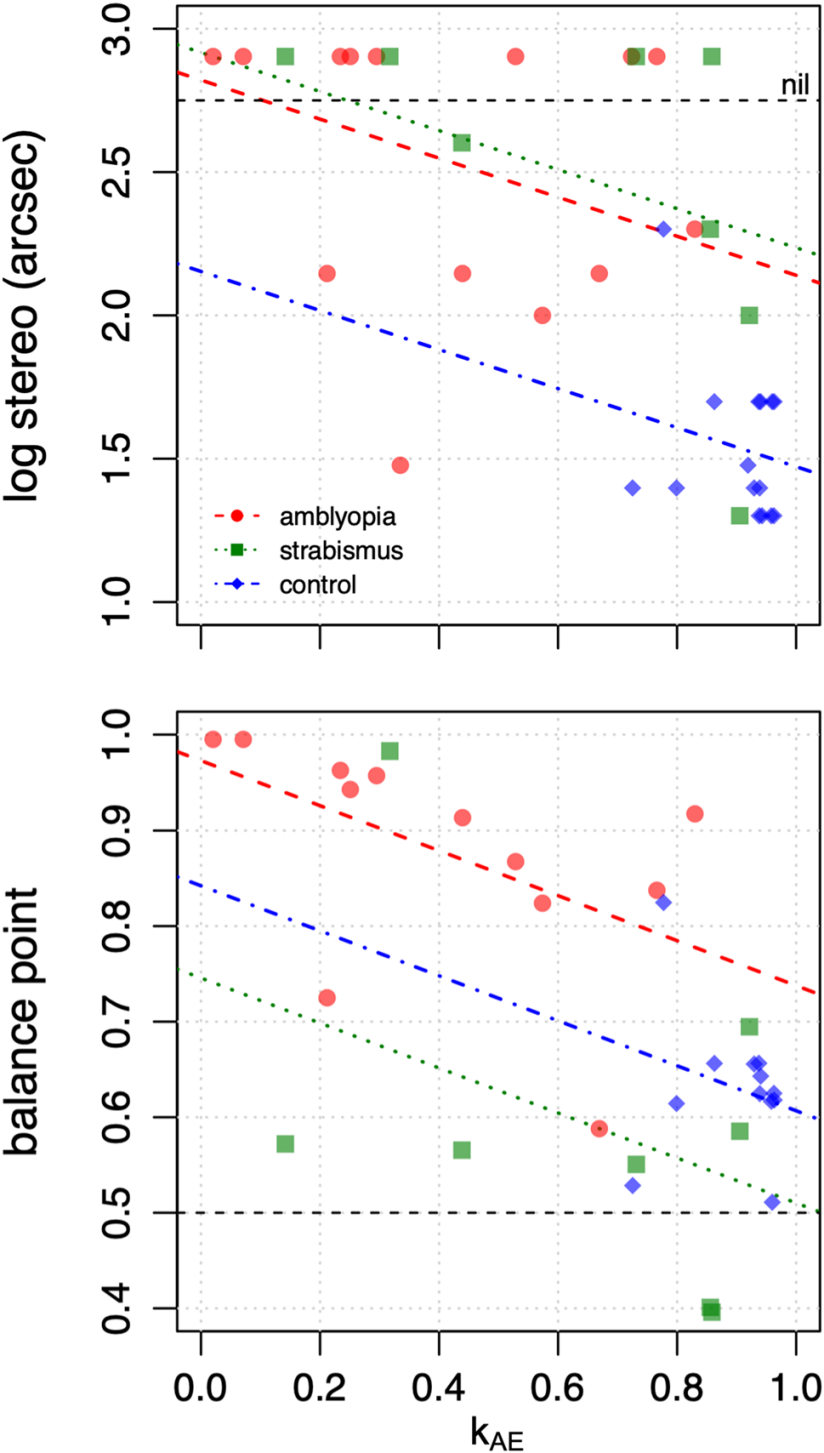
The relationship between k_AE_ and stereoacuity (top) and balance point (bottom) for the regression models described in-text.

Using linear regression, with either Group *or* k_AE_ as predictors, we found that both Group and k_AE_ were significant predictors for all four assessments of visual function: amblyopic eye acuity, stereoacuity, amblyopic eye contrast sensitivity, and interocular balance point (first two columns in Supplementary Table 2). Next we used a nested model F-test to determine whether k_AE_ significantly improved predictions *after* Group had been included as a categorical factor. k_AE_ improved predictions for the two measures of binocular interactions: stereoacuity and interocular balance point. Thus, even after accounting for group membership, k_AE_ was still significantly correlated with stereoacuity and interocular balance point, Fig. 5, such that lower k_AE_ values (obtained with our non-rivalrous stimuli) predicted poorer stereoacuity and a more asymmetric balance point (obtained with a rivalrous dichoptic letter chart) within groups.

The same analysis was performed using our estimated parameters for binocular imbalance, μ_AE_ and μ_FE_ (Supplementary Table 3). None of these parameters significantly predicted performance for any of our visual assessments after controlling for group.

### Reduction of task length

Across participants, joystick lever position *J* was well-correlated during the first and second half of each individual trial (dichoptic trial portion only) within all observers (median *r* = 0.66, SD = 0.12, range = 0.29 to 0.87), indicating that most participants tracked perceived contrast over time with reasonable consistency, see Supplementary Fig. 1.

We were interested in whether we could obtain reliable parameter estimates using shorter trials—something that would be useful in a clinical environment. We re-fit our models using a reduced dataset consisting of 24 trials, each 38 s in length. The correspondence between the original estimates from the full dataset and the estimates for this reduced dataset were high, as shown in Supplementary Fig. 2. There was a near-perfect relationship for k_AE_ (r = 0.98), and σ (r = 0.94). The correlation was smaller, but still reasonable, for μ_AE_ (r = 0.76) and μ_FE_ (r = 0.62). All results of statistical tests between amblyopia and controls led to identical conclusions. Thus, reasonable parameter estimates can be obtained in approximately 15 min of data collection.

### Simulating balance point and masking effects

Using the normalization model of Eqs. (1–3) and the mean best-fitting parameters for each group, we simulated our model’s response across a variety of tasks/stimuli previously examined in the literature. These simulations, based on parameter values estimated under non-rivalrous conditions, consistently *under* predicted the effects of amblyopia for experimental data in rivalrous tasks, consistent with the notion that interocular interactions are larger for rivalrous stimuli.

We began by simulating the dichoptic letter chart balance point^17,21^ collected in our observers. Our simulations predicted a balance point of 0.76 for our amblyopic observers, lower than our experimentally measured balance point of 0.88 (control and non-amblyopic strabismus observers had simulated balance points of 0.51 and 0.64 respectively, compared to obtained values of 0.64 and 0.59). Thus, within the same amblyopic observers, we observed greater interocular suppression for rivalrous letters than for the non-rivalrous gratings used in our main paradigm.

Next, we simulated predictions for the cyclopean phase combination balance point^8^—the perceived phase of a grating produced by presenting opposite phase-shifted sine waves in each eye. This task might be considered ‘semi-rivalrous’—the input in each eye is different, but the two eyes tend to be integrated as a weighted average. For control observers, our simulations predicted that roughly equal contrast would be needed in each eye to produce a phase shift consistent with equal perceived contrast in each eye (equivalent to the balance point), a finding that matched measured values of Ding et al. ^8^. For observers with amblyopia, at the very lowest contrasts, our simulation results roughly matched that of Ding et al.—our model predicted that 2.4× the contrast was needed in the amblyopic eye to reach the balance point. However, at higher contrasts Ding et al. found that a 5–40× increase in contrast was needed to reach balance whereas the effect of contrast of the amblyopic eye was much weaker in our model, which predicted that 2.85× contrast was needed in the amblyopic eye to reach the balance point at the highest contrasts. Thus, for high contrasts, the measured effects of interocular suppression in amblyopia in their rivalrous grating balance task were far larger than those predicted by our model.

Finally, we modeled the elevation in contrast required to see a grating in the amblyopic eye when masked by bandpass noise in the fellow eye^22,23^. For controls, our simulations produced dB threshold elevations of about 0.9 across the two experiments, far lower than the observed range of 8 to 13.7 with rivalrous noise stimuli. For observers with amblyopia, our simulations predicted threshold elevations of about 2.2, whereas published experimental values fall between 13.4 and 17^22–24^. Once again, interocular interactions seem to be larger for rivalrous stimuli than predicted by our model, using parameters estimated using non-rivalrous stimuli.

## Discussion

Here we describe an intuitive, efficient, non-rivalrous task for assessing interocular interactions in which observers continuously report the perceived contrast of dichoptic grating stimuli that are identical, except that stimuli are contrast-modulated independently over time in each eye. Participants were observers with amblyopia, strabismus with equal vision, and controls. We fit a model to observers’ responses that agnostically identified the amblyopic eye and allowed us to estimate attenuation of the amblyopic eye and divisive normalization across the two eyes. We found that observers with amblyopia showed greater attenuation and reduced normalization of the fellow eye by the amblyopic eye compared with controls. Normalization of the amblyopic eye by the fellow eye did not differ significantly between groups.

We found significant monocular signal attenuation (k_AE_) of the amblyopic eye. Although this is consistent with previous findings^20^, it is somewhat surprising for two reasons. First, qCSF estimates of sensitivity at 2 cpd (the spatial frequency of our stimulus) did not show sensitivity deficits as a result of amblyopia; consistent with other studies which find amblyopia primarily impacts mid-to-high spatial frequencies^25–27^. Second, our stimulus contrast was primarily suprathreshold, and monocular contrast perception above threshold in the amblyopic eye is generally near-normal for contrast matching and contrast estimation tasks^28,29^.

One possible explanation for this finding of monocular attenuation for suprathreshold stimuli is that during the trial phases used to estimate monocular attenuation of the amblyopic eye, the fellow eye was open and viewing a mean-luminance screen along with a fusion-lock frame. The presence of a foveal signal (albeit non-overlapping) and/or a mean contrast signal in the fellow eye (as compared to patching one eye) may have resulted in attenuation of the amblyopic eye.

Another possibility is that contrast constancy^30^ may break down in amblyopia when monocular periods of stimulation are embedded in the context of dichoptic stimulation—our periods of monocular stimulation were relatively brief (6–8 s), with no cue to their onset. A loss in suprathreshold sensitivity for contrast-matching under dichoptic conditions has previously been shown^20^, and, similar to our study, could be modeled as a combination of input attenuation and increased suppression.

Thus, *monocular* attenuation, as measured in our study, may be dependent on the previous *binocular* context. This finding is consistent with a variety of studies showing adaptive response normalization across short and long timescales across a wide variety of domains that includes contrast^31–33^. Monocular attenuation in the context of binocular stimulation (conditions more reflective of natural vision) may be larger than under strictly monocular conditions, when the fellow eye is fully occluded, as is often the case for tests used in the clinic to assess amblyopic function.

We found that individual differences in monocular attenuation were significantly related to all four measures of visual function (acuity, stereopsis, contrast sensitivity, and balance point). However, after controlling for group (amblyopia, strabismic with equal vision, or control), only the two measures reflecting binocular interactions—balance point and stereoacuity—remained significantly correlated with attenuation, such that greater attenuation of the amblyopic eye was associated with worse stereoacuity and a more asymmetric balance point. The relationship between attenuation and these two measures may be directly causal. Balance point reflects an interocular contrast ratio, and so is expected to be directly related to perceived contrast. With respect to stereoacuity, reducing the contrast of the image shown to one eye elevates stereoacuity thresholds in control observers^34^, while reducing the contrast of the image sent to the fellow eye improves stereoacuity thresholds in amblyopic observers^35^, consistent with the idea that monocular contrast attenuation interferes with stereoscopic processing.

We saw a significant reduction in the normalization of the fellow eye by the amblyopic eye in amblyopic observers (μ_FE_). In fact, in 4 of our 14 observers with amblyopia we obtained μ_FE_ values at or near zero, indicating no influence at all of the amblyopic eye on the fellow. We did not see significantly different normalization of the amblyopic eye by the fellow eye (μ_AE_), though these estimates varied among observers with amblyopia. This is consistent with converging evidence from a variety of psychophysical results in humans^8,22–24^ and electrophysiological evidence from non-human primates^36^ indicating that amblyopic suppressive imbalance is not driven by excessive suppression of the amblyopic eye by the fellow (as was previously assumed, and is the basis of many traditional therapies), but rather may reflect a weakened ability of the amblyopic eye to exert influence over the fellow eye. This asymmetry in suppression has been shown in dichoptic masking paradigms that either (like our study) used stimuli for which participants did not show monocular deficits^23^, or stimuli that compensated for monocular deficits by increasing the contrast in the amblyopic eye^37^.

Our finding that neither attenuation nor normalization in amblyopes predicted visual acuity (after accounting for group) was somewhat surprising, and implies that visual acuity may not adequately summarize amblyopic function^38^. Our task focused on stimuli at 2 cpd, a spatial frequency where contrast sensitivity is almost unimpaired (at least in our participant group). It seems plausible that attenuation and/or normalization would vary as a function of spatial frequency, as has been shown for rivalrous ^8,17,22,39^ and non-rivalrous stimuli ^24,37^ so these parameter estimates might be more strongly correlated with visual acuity and/or contrast sensitivity when measured using higher frequency stimuli.

Interocular normalization measures (whether from amblyopic to fellow, μ_FE_, or vice versa, μ_AE_) did not predict performance on any of the visual functions we measured, perhaps because most (apart from stereoacuity) were monocular or rivalrous binocular tasks.

Finally, using our normalization model we simulated responses across a variety of tasks and stimuli. Interocular interactions in rivalrous tasks^8,17,21–23^ are consistently larger than predicted by our model, suggesting that the impact of amblyopia may be exaggerated when using rivalrous stimuli as compared to more naturalistic non-rivalrous stimuli.

Collectively, these results are consistent with the intuition that there is likely to be a fundamental difference between the *contrast* *normalization* that occurs when the signals between the two eyes are consistent (which likely reflects a form of weighted neural averaging/integration) and the interocular interactions that occur when the signals between to the two eyes are locally (as occurs for partial occlusions) or wholly rivalrous, which may result in a form of ‘winner take all’ suppression.

It should be emphasized that the model described here does not attempt to explain the full complexity of interocular interactions. It is likely that our simple model would fail to fit data using the same paradigm but orthogonal gratings in each eye: consistent with the observation that models of amblyopia using rivalrous stimuli tend to be more elaborate than the model described here^7,8,13,16,40^. However, our model does succinctly describe interocular interactions under non-rivalrous conditions that favor integration rather than suppression, contributing to a growing literature showing that measurements of amblyopic vision are different for rivalrous gratings than for more naturalistic stimuli^41^.

Our model assumes that attenuation and suppression are constant over time. As a consequence, our model cannot describe the behavior of an observer with equal vision and alternating strabismus (and therefore alternating suppression) who switches fixation during the task; since this would require different parameters for μ during right vs. left eye fixation (e.g. high μ for right and low for left during left eye fixation, and vice-versa). However, model parameters were not substantially different when we restricted the input data to the model to points in time when the contrast difference between the two eyes was less than 0.5, limiting the potential for rivalry. Moreover, examination of rivalrous models, Supplementary Materials, Appendix II, suggests that participants’ percepts could not be explained by models assuming complete alternating suppression, wherein only one eye contributed to the percept at any point in time.

A key advantage of our contrast-perception paradigm is that it can easily be generalized to a wide variety of stimuli: motion, faces, letters, natural scenes, and so on. Although tasks have been developed to assess and compare the effects of amblyopia across a variety of stimulus domains, it can be difficult to generate pairs of tasks which allow one to be confident that differences in parameter estimates are due to differential effects of amblyopia in brain regions associated with different visual features, rather than differences in the stimulus (e.g. moving dots vs. stationary Gabors) or task (e.g. direction of motion vs. orientation judgments). Our general paradigm can be used to compare parameter estimates across stimuli matched for spatiotemporal frequency content, for example: moving vs. counter-phase flickering gratings, faces vs. scrambled faces, or natural vs. wavelet-scrambled movies^42^. Our paradigm is also easily extendable to the neuroimaging domain (either EEG/MEG or MRI), allowing one to directly compare neural and perceptual time series data.

Another advantage of our paradigm is its efficiency. A multiplicity of dichoptic tasks have been developed to quantify how amblyopia affects the relative strength of eye signals^8,12–17,43^. Many of these have been validated in children^21,44,45^, making them useful research and clinical tools for understanding how interocular suppression changes over time as a function of treatment^46–48^. However these paradigms tend to be either extremely time consuming, require visibility matching^39^, or provide a single measure that cannot successfully isolate both attenuation and suppression (for a brief discussion, see^17^). Our paradigm collects rapid continuous adjustment measurements and is highly efficient: with 15–30 min of data collection, it is possible to reliably fit a model with six-to-seven free parameters that reliably describes both attenuation and interocular suppression. Given that most children today have extensive experience using joysticks and gamepads, it seems likely that this task could be modified to measure the impact of amblyopia therapy in relatively young children.

## Methods

This study was approved by the University of Washington’s Institutional Review Board, and carried out in accordance with the Code of Ethics of the Declaration of Helsinki. Informed written consent was obtained prior to conducting the experiments.

### Participants

Participants were recruited from the greater Seattle, WA community. Participants wore their habitual visual correction during the experiment, if needed.

#### Amblyopia and/or strabismus

Ophthalmological histories and diagnoses were confirmed by an ophthalmologist (author KTH) and all participants with amblyopia or strabismus underwent an ophthalmological assessment, including retinoscopy and alignment/cover testing. Observers with ≥ 0.2 logMAR interocular acuity difference were classified as having anisometropic amblyopia in the presence of ≥ 1 D spherical or ≥ 1.5 D astigmatic difference in best-corrected refraction, strabismic amblyopia in the presence of heterotropia at near and/or far, and mixed amblyopia if both criteria were met. Observers with < 0.2 logMAR interocular acuity difference in the presence of heterotropia were classified as *non-amblyopic* *strabismus* *with* *equal* *vision* and are analyzed as a separate group (this group can include both observers with successfully treated amblyopia and observers who had never had amblyopia). In total, 14 participants had amblyopia (*M* age = 50.5 years, *SD* = 19.4, *range* = 18–75); and 8 participants had strabismus with equal vision (*M* age = 40.2 years, *SD* = 18.3, *range* = 18–65). Information about these participants is detailed in Supplementary Table 4. All participants wore their habitual vision correction during testing.

#### Controls

A total of 16 healthy control participants with no history of vision disorder participated in this experiment (*M* age = 34.6 years, *SD* = 16.4, *range* = 18–69). Because we hoped to capture natural variation in typical development in a group of observers wearing their habitual correction, we did not apply any exclusion criteria to controls based on their performance on any visual assessment tasks. Due to disruptions by the COVID-19 pandemic that occurred when we began running this experiment, five control participants do not have data available from all visual assessment tasks. Rather than excluding these participants, we include their available task data, and report when these data are unavailable.

#### Equipment

Participants viewed stimuli presented on a custom-built mirror stereoscope. A forehead and chin rest were used to stabilize head position. Two mirrors angled at 45° and 135° were positioned in front of the participants’ eyes, each reflecting the image of one of two LED-backlit LCD monitors (Philips 328P6AUBREB) positioned to the left and right of the participant at a viewing distance of 1.36 m. Prior to conducting each task, participants carried out a crosshair alignment task to ensure proper image alignment in the stereoscope. Participants with strabismus who experienced difficulty during the alignment task (n = 3) were manually aligned by the experimenter based on their known deviation; because of the relatively large area and low spatial frequency of the stimulus, extremely precise alignment is not required for this task, so we include their data.

Monitors subtended 28.9° × 16.5° and provided the only source of light in the room. Monitors were linearized with minimum and maximum luminance levels of 0.28 cd/m^2^ and 470 cd/m^2^, respectively. Average luminance of the gratings and the mid-grey background was 235 cd/m^2^. This display system was used for contrast sensitivity testing and the main experiment dynamic contrast task.

To make responses, participants used their thumb and forefinger to move the throttle lever of a Thrustmaster brand USB Joystick (Guillemot Corporation SA). This is a small lever on the side of the device that moves smoothly up-and-down, covering a space of approximately two inches (Fig. 2B).

Custom-built MATLAB scripts (R2021a, The Mathworks, Inc.) using the Psychtoolbox extension version 3.0.15^49–51^ were used for stimulus presentation.

### Dynamic contrast task

Each monitor screen showed a 2 cpd grating patch (Gaussian-windowed at 4°; full size 13° × 13°) on a mean-luminance grey background (Fig. 2A). The grating was always presented at an orientation of 90° on the first frame and rotated slowly counter-clockwise at 1°/s for the duration of the trial to minimize adaptation. Gratings in each eye slowly modulated between 0 to 100% contrast in a sinusoidal fashion at the frequencies described below. A black frame (13° × 13°) surrounded the stimulus in each eye, acting as a fusion lock. This frame remained on the screen at full contrast for the entire trial duration as well as during inter-trial rest periods.

Each trial was 62 s long, and consisted of three different phases of stimulation: binocular, dichoptic, and monoptic. Each trial began with 14 s of *binocular* 1/7 Hz contrast modulation, in which the contrast of the two eyes was identical. Most of the remaining 48 s of the trial was *dichoptic*, such that contrast in one eye modulated at 1/6 Hz while the contrast in the other eye modulated at 1/8 Hz. The periods of these sinusoidal modulations synchronized every 24 s, so each modulation pattern was repeated once during each trial (Fig. 2C).

During each trial there was a short phase of *monoptic* stimulation (examples highlighted in grey, Fig. 2C). During this monoptic phase, the contrast modulation in one eye ‘dropped out’ and the stimulus in that eye remained at 0% contrast for a single cycle, while the other eye continued to modulate. These monoptic phases allowed us to measure monocular contrast response functions in the context of previous dichoptic stimulation.

After a trial was finished, participants were shown a screen with the number of trials they had completed so far. Participants could press a joystick button to initiate the next trial, or verbally ask the research assistant to initiate the next trial. Participants conducted a total of 28 trials in a pseudo-randomized order such that all possible combinations of dichoptic presentation (left eye 1/8 Hz and right eye 1/6 Hz; left eye 1/6 Hz and right eye 1/8 Hz) and timing of the monoptic phase (which 1 of 14 possible cycles was dropped) were presented once.

The participant’s task was to adjust the position of a joystick lever to report the contrast of the stimuli on the screen, such that the lowest position indicated 0% contrast, the highest position indicated 100% contrast, and positions in between corresponded to intermediate contrasts. Participants were encouraged to use the full range of positions on the joystick. Participants were told that the contrast would not always be predictable, and it was stressed that they should not try to anticipate what the contrast would be but should instead focus on reporting what they see in the moment and not to worry if there was a small delay in what they were shown and what they reported. We did not give participants specific instructions on what to do with their eyes except to tell them to look in the middle of the screen. The joystick’s position was sampled at 30 Hz.

Prior to conducting the main experiment, participants were trained to use the joystick lever to report perceived contrast. Training consisted of two phases: (1) moving the joystick lever to control the physically-presented binocular contrast on the screen. This allowed participants to become familiar with the range of possible contrast values and their corresponding joystick positions, and (2) practice trials of binocularly-presented contrast modulating at 1/7 Hz for 21 s, where participants moved the joystick to match the perceived contrast. Each trial was followed by visual feedback consisting of a graph showing physical contrast vs. joystick position over time. Once participants were comfortable with training and seemed to be reporting physical contrast with reasonable accuracy as qualitatively judged by the research assistant based on the participant using the lever’s full range, in an sinusoidal pattern similar to the stimulus with a reasonable lag; over a minimum of three training trials), the experiment commenced.

### Modeling of the dynamic contrast task

As described briefly in the “Results” “Dynamic contrast estimation task”, we used a three-stage model fitting procedure to predict joystick position over time as a function of the contrast presented to each eye. The first stage calibrates the relationship between joystick position and binocular perceived contrast, the second stage models monocular attenuation, and the third stage models binocular interactions. This model is adapted from the binocular contrast normalization model described by Moradi and Heeger^11^.

#### Stage 1: Joystick vs. contrast calibration

Our first modeling stage was designed to characterize the relationship between joystick position and presented binocular contrast.

We used the last 10 s of the 14-s binocular trial portion to calibrate the relationship between each observer’s joystick position (J) and binocular contrast over time. This was done by minimizing the mean square error between the calibrated joystick position *Ĵ* and presented contrast *C* as a function of time (*t*).

Where:

4 $$\widehat{J}\left(t\right)=a+b\cdot J(t-d)$$

5 $$MSE=\frac{1}{N}\sum_{t=0}^{N}{\left(\widehat{J}\left(t\right)-C(t)\right)}^{2}$$

*J* is the joystick lever position (0–1), *d* reflects a time delay between stimulus presentation and observer response, and *a* and *b* represent a linear scaling between joystick location and calibrated joystick position *Ĵ*. A penalization function prevented *d* > 4 s, *b* < 0, to avoid behaviorally unrealistic degenerate solutions. Having estimated *d*, *a*, and *b*, these parameters were held fixed for the second two stages of modeling. Runs in which less than 50% of the full joystick range was used by the participant were excluded. Details of calibration accuracy are reported in Supplementary Table 1.

#### Stage 2: monocular attenuation

Next, data from the monoptic phase of each trial were used to estimate the weights k_L_ and k_R_, as follows:

$$\widehat{C}\left(t\right)= \left({k}_{R}{C}_{R}\left(\mathrm{t}\right)+ {k}_{L}{C}_{L}\left(\mathrm{t}\right)\right)/ \mathrm{max}\left({k}_{R}, {k}_{L}\right) \quad (\mathrm{Eq}. 1,\mathrm{ from Results})$$

6 $$MSE=\frac{1}{N}\sum_{t=0}^{N}{\left(\widehat{J}\left(t\right)-\widehat{C}(t)\right)}^{2}$$

$\widehat{C}\left(t\right)$ is the model prediction of perceived contrast,${C}_{R}\left(\mathrm{t}\right)$ and ${C}_{L}(\mathrm{t})$ are the contrasts presented to left and right eyes respectively, and *Ĵ(t)* is the calibrated joystick position over time, as described in Eqs. 4 and 5.

Because this model was only fit to the monoptic phase of the trials, either ${C}_{R}\left(\mathrm{t}\right)$ or ${C}_{L}(\mathrm{t})$ was always 0. We enforced the constraint that $\mathrm{max}\left(\widehat{C}\left(t\right)\right)=1$. The presence of $\mathrm{max}({k}_{R}, {k}_{L})$ in the denominator, meant that this was achieved by finding the best-fitting solution for which either ${k}_{R}$ or ${k}_{L}$ was 1, with the other parameter, which always less than 1, representing relative attenuation in the amblyopic eye.

For control and strabismus with equal vision participants, we defined the eye with k = 1 as the ‘fellow’ eye ${k}_{FE}$, and the eye with k < 1 as the ‘amblyopic’ eye ${k}_{AE}$. Having estimated ${k}_{AE}$ and ${k}_{FE}$, these parameters were held fixed for the third stage of modeling.

#### Stage 3: binocular interactions

We then modeled the remaining dichoptic portions of each trial, during which contrast differed across the two eyes, using a simple three stage model, consisting of attenuation (parameters held fixed, having been estimated in Stage 2), divisive normalization (such that activity from each eye reduced the gain for the other eye), and a summation of signals from the two eyes.

Linear monocular attenuation was modeled using parameters ${k}_{AE}$ and ${k}_{FE}$, using values previously estimated using the monocular portion of each trial, in Eqs. (1) and (6). Since by design ${k}_{FE}$ was set to 1:

7 $${\mathrm{Attenuation}: {A}_{AE}\left(\mathrm{t}\right)= {k}_{AE}{C}_{AE}\left(t\right); A}_{FE}(\mathrm{t})= {C}_{FE}(\mathrm{t})$$

Interocular interactions were modeled using contrast normalization, with ${\upmu }_{AE}$ and ${\upmu }_{FE}$ reflecting the extent to which the signal in one eye is reduced by the signal in the other eye:

8 $${\mathrm{Interocular normalization}: \widehat{C}}_{AE}\left(\mathrm{t}\right)= \frac{{A}_{AE}\left(t\right)}{{\mu }_{AE}{C}_{FE}\left(\mathrm{t}\right)+\sigma }; {\widehat{C}}_{FE}(\mathrm{t})= \frac{{C}_{FE}(t)}{{\mu }_{FE}{A}_{AE}\left(\mathrm{t}\right)+\sigma }$$

A grid search was performed (parameter sweep, 0 ≤ σ ≤ 1; 0 ≤${\upmu }_{AE}$ ≤ 3; 0 ≤ ${\upmu }_{FE}$ ≤ 3) followed by mean squared error minimization to find σ, ${\upmu }_{AE}$, and ${\upmu }_{FE}$﻿. After estimation of σ, ${\upmu }_{AE}$, and ${\upmu }_{FE}$, these parameters were held constant and the value of ${\mathrm{k}}_{AE}$ was re-estimated using the full dataset (i.e. both monoptic and dichoptic phases).

Finally, we assume the final perceived contrast is simply the sum of the outputs of the left and right eyes (no free parameters):

9 $$\mathrm{Linear combination}: \widehat{C}(t)= {\widehat{C}}_{AE}(\mathrm{t})+ {\widehat{C}}_{FE}(t)$$

Thus the parameters *a*, *b*, *d* were estimated based on binocular trial phases, k_AE_, k_FE_, were estimated based on monoptic trial phases, and μ_AE_, μ_FE_, and σ were estimated based on dichoptic trial phases.

### Simulating other task predictions

Using the mean group best fitting parameters from our model it is possible to simulate our model’s response to other tasks and stimuli previously used in the literature. Using the normalization model above we simulated three tasks/stimuli: (1) our balance point data measured using a dichoptic letter chart (the interocular contrast ratio that a participant requires to report seeing the letters in the left and right eye with equal probability), (2) a balance point as estimated by Ding et al., based on the perceived phase of a 2.72 cpd cyclopean sinewave grating produced by presenting opposite phase-shifted sinewaves to each eye^8^, and (3) the elevation in contrast required to see a grating in one eye with a noise mask in the other eye^22,23^.

To simulate balance points, we used function minimization to find the contrast in the amblyopic and fellow eyes that produced equal perceived contrast across both eyes, with the experimental constraint that the contrast across the two eyes summed to 1.

To simulate cyclopean grating balance measurements, the contrast of the non-dominant eye was fixed to reported experimental values^8^, ranging from 0.0075 to 0.96, and function minimization was used to find the contrast of the fellow eye that resulted in equal perceived contrast across both eyes.

To simulate the effects of masking, we first found the perceived contrast that corresponded to the experimental monocular contrast thresholds in the amblyopic (or non-dominant) eye^22,23^. We assumed that a grating in the amblyopic eye would be visible whenever it reached that perceived contrast: and defined this as the *perceived* *contrast* *threshold*. We then fixed the contrast in the fellow eye at the reported experimental contrast of the mask and used function minimization to find the contrast in the amblyopic eye that was necessary to reach the *perceived* *contrast* *threshold*. We quantified the elevation in threshold (TE) produced by the mask as:

10 $$TE=20 \times log10\left({Thr}_{mask}/{Thr}_{no mask}\right).$$

### Assessments of visual function

We carried four assessments of visual function, as follows.

### Visual acuity

High-contrast visual acuity was assessed binocularly and monocularly, while the other eye was occluded, using the Acuity letters (5-letter optotype row) program of the Freiburg Visual Acuity Test (FrACT^52,53^) at a 1.5 m viewing distance. Black letters (0.45 cd/m^2^) were displayed on a white background (480 cd/m^2^) using an iMac with a 5 K Retina display. The best measurable acuity on this set-up was −0.22 logMAR (20/12 Snellen).

Due to disruptions in data collection, acuity data were not collected from three control participants and one non-amblyopic strabismus observer who had 20/20 vision in both eyes according to their most recent optometrist documentation.

### Stereoacuity

Stereoacuity was assessed using the circles portion of the Randot Stereotest (Stereo Optical Co., Inc.). For analysis purposes, participants with nil stereoacuity are assigned a value of 800 arcsec; all analyses are conducting using log10(arcsec) values. Stereopsis data could not be collected from 1 control participant. Eight of 14 participants with amblyopia and 4 of 8 of participants with non-amblyopic strabismus had no measurable stereopsis using the Randot Circles; these participants are assigned a value of 800 arcsec for analysis purposes.

### Contrast sensitivity

Contrast sensitivity (monocular and binocular) was assessed using the Quick CSF method^54^ with 120 trials. Briefly, in this task participants discriminated the orientation of grating stimuli presented at 45° or −45°, which remained on the screen until response. Monocular contrast sensitivity was assessed dichoptically, while the other eye was shown a mid-grey background. This task characterizes several useful descriptors of an individual’s contrast sensitivity function; for simplicity, we used log CSF sensitivity at 2 cpd, and the area under the log CSF curve (AUC) as summary measures of contrast sensitivity.

Data were not available from four control participants and we could not estimate a CSF in two participants with amblyopia. It was not clear whether they were unable to see the stimulus with either eye, or whether they misunderstood the task.

### Interocular balance point

Balance point was assessed using a dichoptic letter chart (implemented as in^21^; also see^17^). Briefly, the contrast of dichoptically-presented band-pass (3 cycles/letter) filtered Sloan letters was controlled using a staircase to determine the interocular contrast ratio a participant required to report seeing the letters in the left and right eye with equal probability. We assessed balance point for three letter sizes: with peak center frequencies of 1, 2, and 4 cpd. Not all observers with amblyopia, particularly those with poor acuity, could conduct this task at the smaller letter sizes. Because of the high correlation among these measures (*r* ≥ 0.73) and the fact that there were no significant differences among them (*p* ≥ 0.31), we took the average balance point of as many measures as each participant was able to conduct. One participant with amblyopia was unable to conduct the task at any letter size (possibly due to amblyopic eye acuity of 1.06 logMAR). Data were not collected from 4 control participants.

### Statistical data analysis

The results of our visual assessment tasks (acuity, stereoacuity, contrast sensitivity and the interocular balance point) and model fits (k_AE,_ μ_AE,_ μ_FE,_ σ, and mean square error in fit) were analysed using t-tests (with Welch correction when appropriate) and ANOVA analyses using group and eye as factors. Effect sizes for pairwise tests are reported using Glass’s ∆ using the control group’s standard deviation for reference. Because of the low sample number of strabismus with equal vision participants (n = 8), these data are qualitatively described; aside from the regression models described below, formal statistics only compare performance between amblyopia and controls.

To determine whether the parameters of our three-stage model could provide predictive information about participants performance on visual assessment tasks we carried out hierarchical linear regression. In step 1 we predicted visual assessment measurements as a function of group membership alone. In step 2 we examined whether the addition of a model parameter as a predictor improved regression predictions. We used a nested F-test to determine if the increase in R^2^ between steps 1 and 2 was statistically significant. All participants were used in this analysis, including strabismus with equal vision.

Statistical tests were carried out using R 4.0.4 (R Core Team, 2021), with the aid of the packages car^55^ (v 3.0–11), rstatix^56^ (v0.7.0), and tidyr^57^ (v1.1.3).

## Supplementary Information

Supplementary Information.

## Data Availability

Data and scripts for analysis are available at the UW’s Vision and Cognition Group github repository, which can be found at: https://github.com/VisCog.
