# Supplementary material for: Characterizing amblyopic perception under non-rivalrous viewing conditions
Source: Sci Rep. 2023 May 17;13:7993. doi: 10.1038/s41598-023-31301-8 (PMC10189719; doi:10.1038/s41598-023-31301-8)
Supplement: Supplementary file 1 — Supplementary Information. [file 41598_2023_31301_MOESM1_ESM.pdf]

SUPPLEMENTARY MATERIALS

Supplementary Tables

Supplementary Table 1. Stage 1 fits: Joystick calibration parameters. Mean (SD) values are shown. Statistical tests compare control vs. amblyopia groups.

	All participants	Control	Amblyopia	Strabismus with equal acuity
d (in seconds)	0.76 (0.39)	0.77 (0.31)	0.82 (0.28)	0.65 (0.27)
		$t(28) = 0.42$, $p = 0.68$		
a	0.17 (0.19)	0.12 (0.14)	0.28 (0.23)	0.07 (0.14)
		$t(21.3) = 2.18$, $p = 0.040$		
b	1.386(0.34)	1.28 (0.27)	1.51 (0.42)	1.27 (0.21)
		$t(21.9) = 1.75$, $p = 0.093$		
MSE	0.0016 (0.0013)	0.0012 (0.0009)	0.003 (0.0014)	0.0000 (0.0011)
		$t(19.3) = 1.59$, $p = 0.13$		

Supplementary Table 2. Linear regression results using Group and/or k_{AE} as predictors.

	Group alone	*k_{AE} alone*	*k_{AE} after Group*	*Regression models (control group set as reference group)*
Acuity	**$F(2,31) = 32.74$, $p < 0.0001$ ***	**$F(1,32) = 16.40$, $p < 0.0001$ ***	$F(1,30) = 0.77$, $p = 0.39$	
Stereoacuity (log10)	**$F(2,34) = 22.51$, $p < 0.0001$ ***	**$F(1,35) = 27.24$, $p < 0.0001$ ***	**$F(1,33) = 4.37$, $p = 0.044$ ***	$b_0 = 2.82$ (SE = 0.18), $t(33) = 15.92$, $p < 0.0001$; $b_{amblyopia} = 0.10$ (SE = 0.20), $t(33) = 0.49$, $p = 0.63$; $b_{strabismus} = -0.67$ (SE = 0.22), $t(33) = 3.05$, $p = 0.0045$; $b_{kAE} = -0.68$ (SE = 0.33), $t(33) = 2.09$, $p = 0.0045$; $R^2 = 0.62$, $R^2_{adj} = 0.59$, $F(3,33) = 17.95$, $p < 0.0001$.
Contrast sensitivity	**$F(2,29) = 8.50$, $p = 0.0012$ ***	**$F(1,30) = 7.59$, $p = 0.0099$ ***	$F(1,28) = 0.83$, $p = 0.37$	
Interocular balance point	**$F(2,29) = 16.1$, $p < 0.0001$ ***	**$F(1,30) = 19.68$, $p = 0.00011$ ***	**$F(1,28) = 6.12$, $p = 0.020$ ***	$b_0 = 0.97$ (SE = 0.05), $t(28) = 18.93$, $p < 0.0001$; $b_{amblyopia} = -0.23$ (SE = 0.06), $t(28) = 3.92$, $p = 0.0005$; $b_{strabismus} = -0.13$ (SE = 0.07), $t(28) = 1.97$, $p = 0.059$; $b_{kAE} = -0.24$ (SE = 0.10), $t(28) = 2.47$, $p = 0.020$; $R^2 = 0.61$, $R^2_{adj} = 0.57$, $F(3,28) = 14.67$, $p < 0.0001$.

*Note. * Statistically significant multiple regression model.*

Supplementary Table 3. Linear regression results using μ_{AE} or μ_{FE} as predictors.

	μ_{AE} *alone*	μ_{AE} *after Group*	μ_{FE} *alone*	μ_{FE} *after Group*
Acuity (amblyopic eye)	$F(1,31) = 3.00$, $p = 0.093$	$F(1,29) = 0.28$, $p = 0.60$	$F(1,32) = 5.60$, $p = 0.024$ *	$F(1,30) = 0.00$, $p = 0.94$
Stereoacuity (log10)	$F(1,34) = 4.75$, $p = 0.036$ *	$F(1,32) = 1.43$, $p = 0.24$	$F(1,35) = 13.15$, $p = 0.0009$ *	$F(1,33) = 0.07$, $p = 0.79$
Contrast sensitivity (amblyopic eye AUC)	$F(1,29) = 4.06$, $p = 0.053$	$F(1,27) = 0.96$, $p = 0.34$	$F(1,30) = 3.75$, $p = 0.062$	$F(1,28) = 0.67$, $p = 0.42$
Interocular balance point	$F(1,29) = 1.28$, $p = 0.27$	$F(1,27) = 0.01$, $p = 0.93$	$F(1,30) = 2.72$, $p = 0.11$	$F(1,28) = 0.05$, $p = 0.83$

*Note. * Statistically significant multiple regression model.*

Supplementary Table 4. Clinical details of participants with amblyopia and/or strabismus.

ID	Subtype	Age	Acuity			Randot Circles (arcsec)	Treatment history	Refractive correction worn for testing
			BE	AE	FE			
A1	aniso	25	-0.06	0.23 (R)	-0.10	30	Patching	R: +3.00 +1.00 x83<br>L: -3.00
A2 §	aniso	41	-0.05	0.60 (L)	-0.12	140	Patching	R: +3.25 +0.50 x10<br>L: +4.25 +0.75 x165
A3 §	aniso	46	0.06	0.56 (L)	0.36	140	No patching	R: -6<br>L: none ‡
A4	aniso	71	-0.11	0.23 (L)	-0.07	100	Patching	R: +3.25 +1.5 x166<br>L: +5.25 +1.0 x135
A5 *	aniso + strab	18	0.11	0.33 (R)	0.09	200	Patching; No Sx	R: +5.50 -1.75 x170<br>L: +4.75 -1.25 x180
A6	aniso + strab	23	-0.21	1.06 (R)	-0.21	Nil	Patching; No Sx	R: +1.0 +0.25 x27<br>L: -1.75 +0.25 x99
A7	aniso + strab	42	-0.11	0.55 (R)	-0.22	Nil	Patching; 7 Sx	R: -5.5 +3.25 x82<br>L: -7.0 +2.50 x82
A8	aniso + strab	61	0.02	0.18 (R)	-0.01	Nil	Patching; 1 Sx	R: +1.50<br>L: +5.50 ‡
A9 ¶	aniso + strab	70	-0.11	0.79 (L)	-0.13	Nil	Patching; 2 Sx	R: -1.5 +1.25 x145<br>L: plano
A10	aniso + strab	72	0.51	0.92 (R)	0.67	Nil	Patching; 3 Sx	R: plano<br>L: -2.50 +1.75 x155
A11	aniso + strab	75	0	0.33 (L)	-0.03	Nil	Patching; No Sx	R: -1.00 +2.00 x15<br>L: plano +1.25 x155
A12 §	strab	46	0.02	0.95 (R)	0.04	Nil	No patching; no Sx	R: +4.75 +0.50 x104<br>L: +4.00
A13 §	strab	51	-0.04	0.96 (L)	-0.06	Nil	Patching; 4 Sx	none
A14	strab	66	0.09	0.55 (L)	0.19	140	Patching; 2 Sx	R: -0.5 +0.5 x97<br>L: -0.75 +1.00 x103
S1	strab (equal vision)	18	0.05	0.13 (L)	0.02	100	Patching; 3 Sx	R: -5.00 +2.25 x85<br>L: -3.75 +1.75 x85
S2 §	strab (equal vision)	22	-0.08	0.00 (L)	-0.04	Nil	Patching; 3 Sx	R: -6.5<br>L: -6.75 ‡
S3	strab (equal vision)	22	-0.02	0.08 (L)	0.05	Nil	Patching; 2 Sx	R: +4.00<br>L: +4.75 ‡
S4	strab (equal vision)	36	-0.19	-0.08 (R)	-0.11	20	No patching; no Sx	R: -2.25<br>L: -2.25 ‡

S5	strab (equal vision)	50	-0.16	-0.14 (L)	-0.06	Nil	Patching; 3 Sx	R: -1.75 +0.75 x95<br>L: -2.00 +0.25 x65
S6	strab (equal vision)	50	-0.03	0.01 (L)	-0.02	400	No patching; no Sx	R: -8.75 +0.50 x170<br>L: -10.00 + 1.50 x15
S7 §	strab (equal vision)	59	N/A†	N/A†	N/A†	200	No patching; no Sx	R: -0.50<br>L: -0.25 ‡
S8	strab (equal vision)	65	0.15	0.20 (L)	0.22	Nil	Patching; 2 Sx	R: -0.5 +0.75 x2<br>L: +0.5 +0.5 x7

Note. Aniso = anisometropia; strab = strabismus; BE = both eyes; AE = amblyopic eye; FE = fellow eye. The amblyopic eye of participants in the non-amblyopic strabismus with equal vision group reflects the eye assigned as amblyopic, as described in the main text. Unless noted otherwise, participants' residual undercorrection was <1 D spherical equivalent anisometropia and <1 D anisoastigmatism. *Participant was assigned k=1 for both the amblyopic and fellow eyes (see text Section 4.3.2). †No acuity data available due to COVID-19 disruption. ‡Wearing contact lenses. §1.0 – 1.5 D uncorrected spherical equivalent anisometropia. ¶4.5 D undercorrected spherical equivalent anisometropia and 1.5 D uncorrected anisoastigmatism.

Supplementary Figures

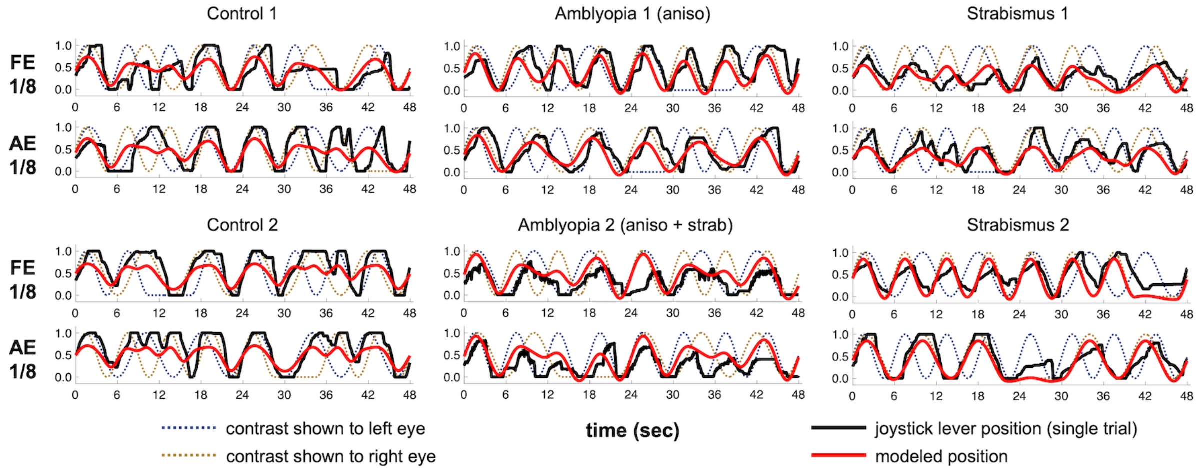

Supplementary Figure 1. Example individual time-courses for single trials with full model fits, for typical participants from the control (left column), amblyopia (middle column) and non-amblyopic strabismus with equal vision (right column) groups. Participant 1 from each group corresponds to the participants shown in Figure 2C. For each participant, the top trace shows one trial where the participant's "fellow" eye received 1/8 Hz contrast modulation and the "amblyopic" eye received 1/6 Hz; the bottom trace shows vice versa. The top and bottom observers with amblyopia correspond to A2 and A5, respectively, from Supplementary Table 4; the top and bottom observers with strabismus correspond to S5 and S8.

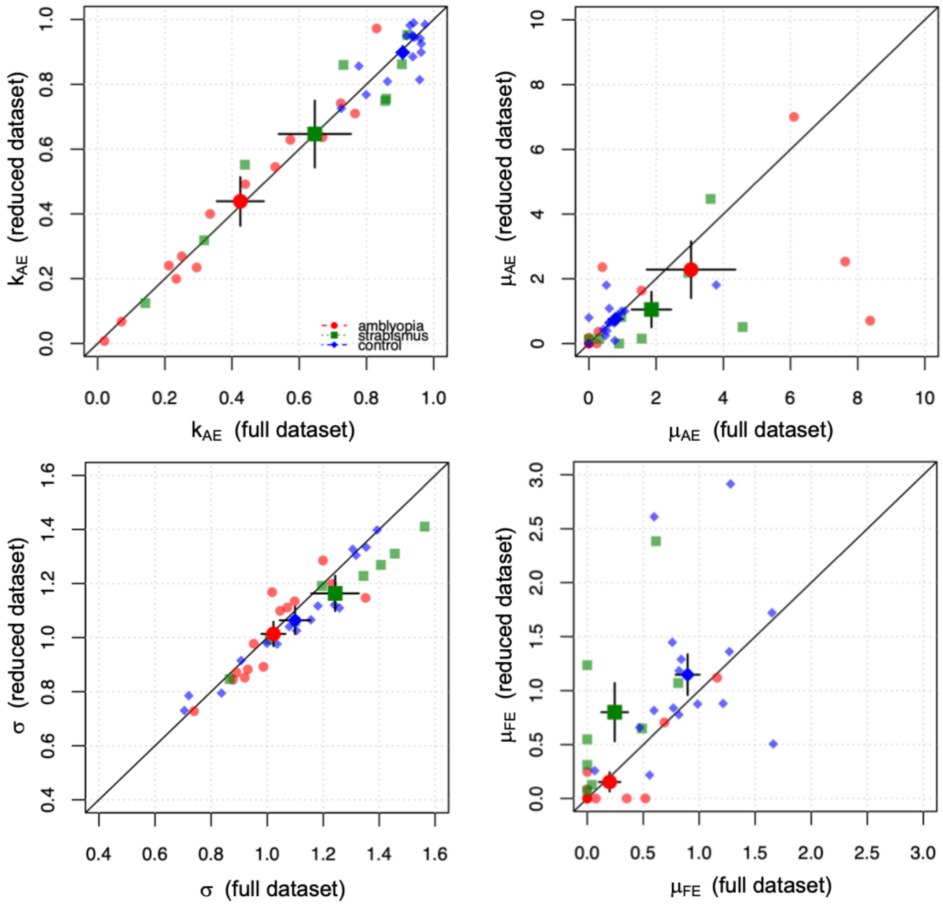

Supplementary Figure 2. The relationship between the original estimates based on the full dataset (x-axis) and the estimates obtained from the reduced dataset (y-axis). Mean values and single standard errors are shown with the larger symbols (some error bars are too small to be seen). Individual data points are shown with the smaller symbols. The diagonal line represents unity.

Supplementary Data

Data and scripts for analysis are available at the UW's Vision and Cognition Group github repository, which can be found at: https://github.com/VisCog

APPENDIX I: COMPARISON TO ALTERNATIVE MODELS

Here we briefly describe comparison models. In the equations below our choices of parameter names/symbols are designed to highlight correspondences between our implementation of attenuation and normalization and the parameterizations used by alternative models.

Attenuation + Normalization

The model used in our paper, was adapted from Moradi & Heeger (2009)'s binocular normalization model by allowing parameter weights for the left and right eye to be estimated independently.

$$\hat{C}(\mathrm{t}) = \frac{k_{AE} C_{AE}(t)}{\mu_{AE} C_{FE}(\mathrm{t}) + \sigma} + \frac{C_{FE}(\mathrm{t})}{\mu_{FE} \cdot k_{AE} C_{AE}(t) + \sigma} \quad \text{(A1)}$$

Mean Joystick

The mean calibrated joystick response over the entire run.

$$\hat{C}(\mathrm{t}) = \overline{\hat{J}(t)} \quad \text{(A2)}$$

Stimulus Average

The average of the stimuli presented to each eye over time.

$$\hat{C}(\mathrm{t}) = {(C_{AE}(\mathrm{t}) + C_{FE}(\mathrm{t}))}/{2} \quad \text{(A3)}$$

Weighted Stimulus Average

Weighted average of the stimuli presented to each eye over time.

$$\hat{C}(\mathrm{t}) = w.C_{AE}(\mathrm{t}) + (1-w).C_{FE}(\mathrm{t}) \quad \text{(A4)}$$

Attenuation + Normalization over Time

This model assumes that normalization, rather than being instantaneous, is based on a temporal average of the stimulus contrast across both eyes.

$$\hat{C}(\mathrm{t}) = \frac{k_{AE} C_{AE}(t)}{\mu_{AE} \varepsilon_{FE}(\mathrm{t}) + \sigma} + \frac{C_{FE}(\mathrm{t})}{\mu_{FE} \cdot k_{AE} \varepsilon_{AE}(t) + \sigma} \quad \text{(A5)}$$

Our model of temporal averaging was based on Ding et al. (Ding & Sperling, 2006; Ding, Klein, & Levi, 2013): ε_{FE} and ε_{AE} represents the contrast in each eye over time passed through a temporal gamma filter consisting of a cascade of five exponential linear filters with time constant τ.

Ding et al. 2013

Also called the DSLK model; used to explain phase shifts created by presenting gratings of different contrast and phase in each eye.

$$\hat{C}(\mathrm{t}) = \frac{1 + \mu_{AE} \cdot \varepsilon_{AE}(t)}{1 + \mu_{AE} \cdot \varepsilon_{AE}(\mathrm{t}) + \mu_{FE} \cdot \varepsilon_{RE}(\mathrm{t})} . k_{AE} . C_{AE}(\mathrm{t}) + \frac{1 + \mu_{FE} \cdot \varepsilon_{FE}(t)}{1 + \mu_{AE} \cdot \varepsilon_{AE}(\mathrm{t}) + \mu_{FE} \varepsilon_{RE}(\mathrm{t})} . k_{AE} . C_{AE}(\mathrm{t}) \quad \text{(A6)}$$

This model was implemented either using a fixed $\tau = 50$ ms (based on Ding & Sperling, 2006) or allowing τ to vary.

Baker et al.

The two-stage model of contrast gain control (Baker, Meese, & Georgeson 2007; Baker, Meese, & Hess, 2008), previously used to predict contrast matching and contrast discrimination with gratings of different contrast but the same orientation presented in each eye. μ_{FE}, μ_{AE}, and σ were constrained to be positive.

$$\hat{C}(\mathrm{t}) = \frac{C_{AE}(t)^k}{C_{AE}(t)+\mu_{AE}\,C_{FE}(t)+\sigma} + \frac{C_{FE}(t)^k}{\mu_{FE}C_{AE}(t)+C_{FE}(t)+\sigma} \quad \text{(A7)}$$

Model comparisons

For models without free parameters (Mean Joystick, Stimulus Average) we calculated the mean squared error (MSE) between the model prediction, $\hat{C}(t)$, and the calibrated joystick position over time, $\hat{J}(t)$ for each participant, across all runs.

For models with free parameters (Weighted Stimulus Average, Attenuation + Normalization, Ding et al. 2013, Baker et al. 2013) we estimated the mean and the standard deviation of the MSE between the model prediction, $\hat{C}(t)$, and the calibrated joystick position over time, $\hat{J}(t)$) using k-fold cross-validation with 4 folds. For each fold, 20-21 runs were used as the training set, and 6-7 runs as the test set. Both the monocular and the dichoptic phases of each run were included in model fitting and testing.

Using the MSE for each subject, we made pair-wise comparisons of each alternative model fit with the fit of our Attenuation + Normalization model, using a two-factor ANOVA with group (amblyopia, strabismus with equal vision, and controls) and model (Attenuation + Normalization, and comparison model) as fixed factors. Table A1 and Figure A1 show data and statistical comparisons between the Attenuation and Normalization model and all other models.

For our data the Attenuation + Normalization model of our paper performs as well as or better than these alternative models. However it is important to note that our stimuli were designed to efficiently measure attenuation and normalization under non-rivalrous conditions, *not to differentiate between different models*. For the stimuli used in our experiment, most of the models we examined make predictions of similar accuracy. The poor performance of the DSKL model, which was designed to capture a very wide range of stimulus conditions, is likely due to over-fitting as a result of our stimulus conditions being relatively restricted.

Appendix Table A1. Statistical comparison of alternative models with the Attenuation and Normalization model. A = amblyopic group, S = Strabismus group, C = Normally sighted controls.

	Mean Error (SD)	Mean Error by Group (SD)	Model Main effect	Group Main effect	Model x Group interaction
Attenuation + Normalization[1]	0.096 (0.053)	A: 0.111 (0.064)<br>S: 0.090 (0.043)<br>C: 0.087 (0.045)			
Mean Joystick[2]	0.189 (0.075)	A: 0.222 (0.095)<br>S: 0.170 (0.050)<br>C: 0.171 (0.057)	$F(1,35) = 220.08$, $p < 0.0001$ *	$F(2,35) = 1.81$, $p = 0.18$	$F(2,35) = 2.82$, $p = 0.073$
Stimulus Average[2]	0.150 (0.080)	A: 0.150 (0.089)<br>S: 0.120 (0.058)<br>C: 0.147 (0.082)	$F(1,35) = 48.41$, $p < 0.0001$ *	$F(2,35) = 0.89$, $p = 0.42$	$F(2,35) = 0.89$, $p = 0.42$
Weighted Stimulus Average[1]	0.135 (0.079)	A: 0.137 (0.085)<br>S: 0.110 (0.062)<br>C: 0.146 (0.082)	$F(1,35) = 23.5$, $p < 0.0001$ *	$F(2,35) = 0.41$, $p = 0.67$	$F(2,35) = 2.74$, $p = 0.078$
Attenuation + Normalization over time[1]	0.104 (0.053)	A: 0.115 (0.064)<br>S: 0.090 (0.044)<br>C: 0.101 (0.047)	$F(1,35) = 23.38$, $p < 0.0001$ *	$F(2,35) = 0.7$, $p = 0.50$	$F(2,35) = 5.58$, $p = 0.008$ *
Ding et al. 2013, $\tau = 50$[1]	0.504 (0.160)	A: 0.486 (0.160)<br>S: 0.430 (0.133)<br>C: 0.555 (0.164)	$F(1,35) = 324.73$, $p < 0.0001$ *	$F(2,35) = 1.03$, $p = 0.37$	$F(2,35) = 3.05$, $p = 0.060$
Ding et al. 2013, τ a free parameter[1]	0.502 (0.157)	A: 0.483 (0.154)<br>S: 0.430 (0.132)<br>C: 0.553 (0.163)	$F(1,35) = 332.17$, $p < 0.0001$ *	$F(2,35) = 1.04$, $p = 0.36$	$F(2,35) = 3.16$, $p = 0.055$
Baker et al. 2013[1]	0.105 (0.057)	A: 0.129 (0.069)<br>S: 0.100 (0.045)<br>C: 0.089 (0.045)	$F(2,35) = 1.51$, $p = 0.24$	$F(2,35) = 1.51$, $p = 0.24$	$F(2,35) = 8.47$, $p = 0.001$ *

[1] mean k-fold cross-validation error across 4 folds; [2]mean MSE error across all runs

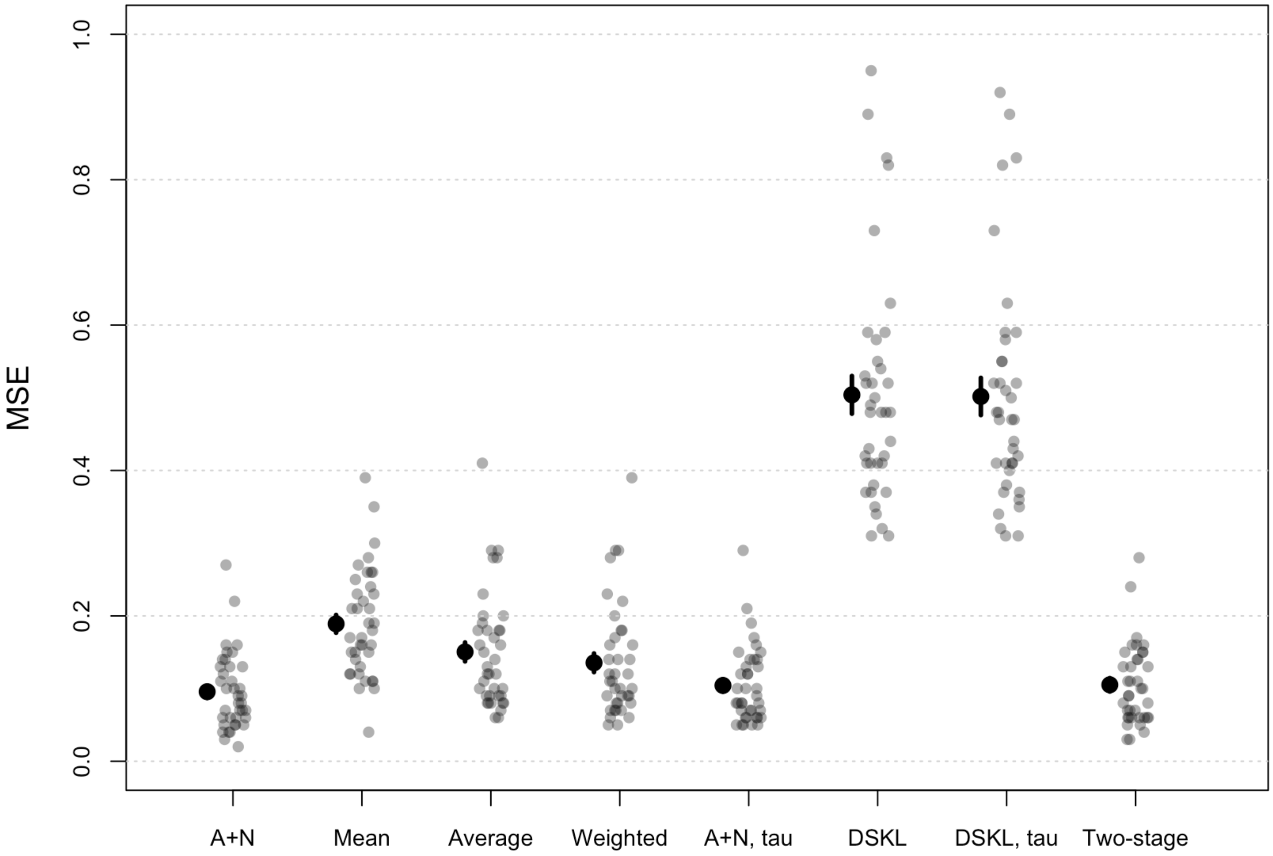

Appendix Figure A1. Mean and standard deviation of MSE error for each participant across different models (corresponding to Table A1). Error bars show the standard deviation across folds (for models with free parameters, MSE was estimated using k-fold validation; see Table A1 footnote). In some cases the error bars are smaller than the symbol size.

References

Baker, DH, Meese, TS, Georgeson, MA. 2007. Binocular interaction: contrast matching and contrast discrimination are predicted by the same model. Spat Vis 20:397–413.

Baker, DH, Meese, TS, Hess, RF. 2008. Contrast masking in strabismic amblyopia: attenuation, noise, interocular suppression and binocular summation. Vision Res 48:1625–1640.

Ding, J, Klein, SA, Levi, DM. 2013. Binocular combination in abnormal binocular vision. J Vis 13:14.

Ding, J, Sperling, G. 2006. A gain-control theory of binocular combination. Proc Natl Acad Sci U S A 103:1141–1146.

Moradi, F, Heeger, DJ. 2009. Inter-ocular contrast normalization in human visual cortex. J Vis 9:13.1–1322.

APPENDIX II: SWITCHING MODELS

Our model (and the models above) assume that attenuation and suppression are constant over time and cannot describe the behavior, for example, of an observer with alternating strabismus who switches which eye is suppressed over time.

Stimulus Maximum

The simplest switching model is to assume that the percept is dominated over time by whichever's eye has the stimulus with maximum contrast.

$$\hat{C}(\mathrm{t}) = \max(\ C_{AE}(\mathrm{t}),\ \ C_{FE}(\mathrm{t})) \qquad \text{(A8)}$$

Rivalry

A second simple rivalry model is to assume that observers switch between eyes over time in an unpredictable way, modeled as:

$$\hat{C}(\mathrm{t}) = E(t).\, C_{AE}(\mathrm{t}) + (1 - E(t)).\, C_{FE}(\mathrm{t})) \qquad \text{(A9)}$$

E is a binary vector of length t, describing which eye is suppressed at each moment in time. E(t) was set to 1 when the amblyopic eye had a contrast value that was closer to $\hat{J}(t)$ (the position of the joystick) than the fellow eye at a given moment in time, and 0 when the fellow eye had a contrast value closer to $\hat{J}(t)$ than the amblyopic eye.

Dual Mean Null model

It is nontrivial to compare the performance of the rivalry model to other models: it has far more free parameters than any of the standard models of binocular integration described above, and E(t) cannot be generalized across different runs, excluding a cross-validation approach.

The traditional statistical 'null model' is the grand mean. The natural extension of this 'null' model, where switching over time occurs, is to assume that the perceived contrast of participants switches between two fixed values over time, with these values set as the average of joystick responses above and below the zero mean, such that:

$J_{below} = \overline{\hat{J}(t, J(t) < 0)}$,

$J_{above} = \overline{\hat{J}(t, J(t) > 0)}$, respectively.

E(t) was set to 1 when $\hat{J}(t) - J_{above} < \hat{J}(t) - J_{below}$, and 0 otherwise.

Thus, for this model:

$$\hat{C}(\mathrm{t}) = E(t).J_{above} + (1 - E(t)).J_{below} \qquad \text{(A10)}$$

Model comparisons

Using each model's mean MSE for each subject, we made pair-wise comparisons between the Attenuation + Normalization model (eq. A1 above) vs. Stimulus Maximum model, and the Rivalry model vs. Dual Mean Null model. For both comparisons we used a two-factor linear mixed model ANOVA with group (amblyopia, strabismus with equal vision, and controls) as a random effect factor. Appendix Table A2 and Appendix Figure A2 show data and statistical comparisons.

Our Attenuation + Normalization model performs significantly better than the Stimulus Maximum model, and the Dual-Mean outperformed the Rivalry model. Although it is impossible to exclude the possibility that there were fluctuations over time in the relative weight accorded to each eye, these results suggest that participants' percepts could not be explained by models assuming complete alternating suppression (wherein only one eye contributed to the percept at any point in time). It is possible that future work could include an elaborated version of the Attenuation + Normalization model that assumes that the observers fluctuate between left and right eye dominant states over time.

Table A2. Statistical comparison of alternative models with the Attenuation and Normalization model. A = amblyopic group, S = Strabismus group, C = Normally sighted controls.

	Mean Error (SD)	Mean Error by Group (SD)	Model Main effect	Group Main effect	Model x Group interaction
Attenuation + Normalization[1]	0.096 (0.053)	A: 0.111 (0.064)<br>S: 0.090 (0.043)<br>C: 0.087 (0.045)	$F(1,35) = 81.47$, $p < 0.0001$ *	$F(2,35) = 2.99$, $p = 0.063$	F(2,35) = 12.62, $p < 0.0001$ *
Stimulus Maximum[2]	0.135 (0.068)	A: 0.174 (0.076)<br>S: 0.130 (0.056)<br>C: 0.101 (0.046)			
Rivalry[1]	0.068 (0.049)	A: 0.081 (0.062)<br>S: 0.060 (0.027)<br>C: 0.063 (0.044)	$F(1,35) = 12.37$, $p = 0.001$ *	$F(2,35) = 1.28$, $p = 0.29$	$F(2,35) = 0.15$, $p = 0.86$
Dual-Mean Null Model[1]	0.052 (0.026)	A: 0.063 (0.033)<br>S: 0.040 (0.014)<br>C: 0.046 (0.023)			

[1] mean k-fold cross-validation error across 4 folds; [2]mean MSE error across all runs

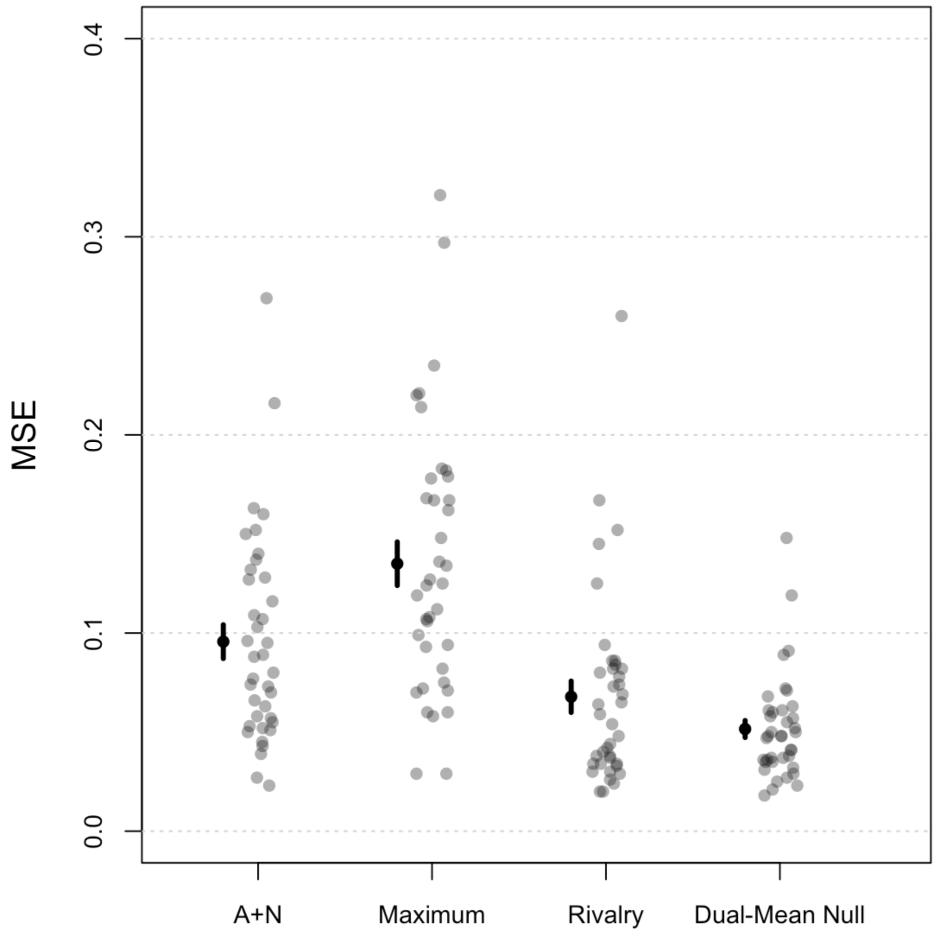

Figure A2. Mean and standard deviation of MSE error for each participant across different models. Error bars show the standard deviation across folds for the Attenuation + Normalization model; the remaining show MSE error in model fit. In most cases the error bars are smaller than the symbol size.
